# Ultrafast CO_2_ Capture from Dilute Streams in Quasi-Equipotential Pores of Metal–Organic Frameworks

**DOI:** 10.1021/acsami.5c05994

**Published:** 2025-07-09

**Authors:** Siriporn Kosawatthanakun, Poobodin Mano, Pawan Boonyoung, Nadhita Chanchaona, Kittipong Chainok, Suwadee Jiajaroen, Supaporn Nualyai, Kajornsak Faungnawakij, Supawadee Namuangruk, Bunyarat Rungtaweevoranit

**Affiliations:** † National Nanotechnology Center (NANOTEC), National Science and Technology Development Agency (NSTDA), Pathum Thani 12120, Thailand; ‡ National Metal and Materials Technology Center (MTEC), National Science and Technology Development Agency (NSTDA), Pathum Thani 12120, Thailand; § Thammasat University Research Unit in Multifunctional Crystalline Materials and Applications (TU-MCMA), Faculty of Science and Technology, Thammasat University, Pathum Thani 12121, Thailand

**Keywords:** CO_2_ capture, metal−organic frameworks, adsorption kinetics, Zn-based-MOF, recyclability

## Abstract

Solid sorbents capable of capturing CO_2_ at particularly low concentrations with rapid kinetics are crucial for effective CO_2_ capture. Here, we report ZnDTZ, a metal–organic framework (MOF) designed with an optimized pore size and functionalized pore surfaces tailored for CO_2_ adsorption. ZnDTZ MOF exhibits exceptional CO_2_ capture performance, achieving an uptake of 1.97 mmol/g at 303 K and 0.05 bar. The spatial distribution of CO_2_ molecules and their interactions with the MOF are revealed by a combination of *in situ* Fourier transform infrared (FTIR) spectroscopy, density functional theory (DFT) calculations, and grand canonical Monte Carlo (GCMC) simulations which indicate that the molecules are stabilized within the pores through multiple binding sites, significantly enhancing adsorption efficiency at low concentrations. Remarkably, ZnDTZ shows unusually fast CO_2_ adsorption kinetics compared to the current benchmark MOF adsorbent, CALF-20, despite similarities in chemical composition. A comprehensive analysis of adsorption kinetics and DFT calculations reveals that the enhanced performance arises from barrierless diffusion within the pores, enabled by the equipotential surface of ZnDTZ, achieved through the contiguous arrangement of the adsorption sites. Notably, ZnDTZ demonstrates excellent recyclability, maintaining stable performance over 200 adsorption–desorption cycles.

## Graphical abstract

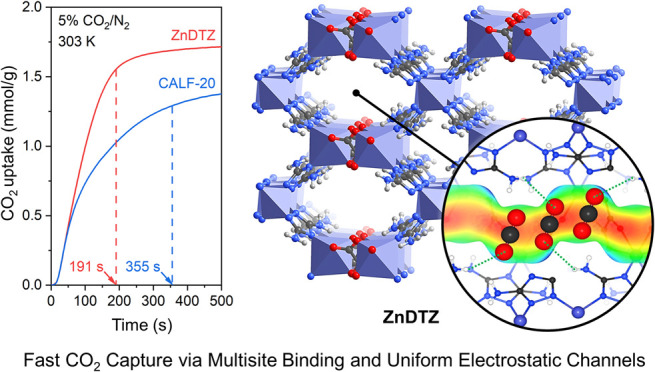

## 1 Introduction

Carbon dioxide (CO_2_) capture is expected to play a pivotal role in achieving carbon neutrality by capturing CO_2_ from dilute streams, such as flue gas, and concentrating it into higher–purity feeds for subsequent utilization, whether as a feedstock for chemical transformations or for long-term storage.^1–3^ The compositional diversity of emission sources presents a significant challenge, requiring CO_2_ capture technologies to accommodate varying concentrations of CO_2_, ranging from 3–5% in natural gas power plants^4–6^ to 10–15% in coal–fired power plants,^7–9^ as well as emissions from other sources.^10,11^ This variability necessitates the development of advanced materials capable of efficiently adsorbing CO_2_ at concentrations relevant to these emission sources.

Solid sorbents have emerged as promising candidates to overcome the high energy penalties associated with regenerating traditional amine-based solvents.^12–16^ Among these, metal–organic frameworks (MOFs) are particularly attractive due to their highly tunable structures, which can be precisely engineered to optimize CO_2_ binding interactions.^17–19^ For practical CO_2_ capture, materials should exhibit sufficient capacities at CO_2_ partial pressures relevant to the target applications. Meanwhile, at CO_2_ concentrations below 5%, the interaction between the MOFs and CO_2_ must be strong enough to ensure sufficient adsorption capacity.^4,20^ Generally, MOFs designed for low–concentration CO_2_ capture often incorporate chemisorptive sites, such as open metal sites,^21–26^ alkylamine functionalities,^27–32^ or anionic sites,^33,34^ or utilize pore confinement effects to enhance adsorption performance.^4,35,36^

The majority of the work in this area focuses on the CO_2_ adsorption properties under equilibrium conditions, which show significant promise for CO_2_ capture applications. An equally critical metric, yet typically overlooked, is adsorption kinetics, which directly impact the productivity of the sorbents by governing the number of CO_2_ capture cycles achievable within a given time frame. This parameter becomes significantly important when integrating MOFs into practical devices such as temperature swing adsorption (TSA),^37–39^ pressure swing adsorption (PSA),^40–43^ or membrane separation systems,^44–46^ where kinetics can impact unit footprint, cost efficiency, and operational effectiveness.

Here, we present the synthesis and characterization of ZnDTZ, a MOF with the chemical formula [Zn­{3,5-diamino-1,2,4-triazole (DTZ)}­{oxalate}_0.5_]·H_2_O, prepared both as single crystals and as a powder through a scalable, aqueous, and ambient pressure synthesis method. Such structure has been reported only recently as NICS-24 ^47^ and IISERP-MOF36.^48^ However, the information on CO_2_ adsorption sites and their adsorption kinetics that is unique to this MOF has not been thoroughly identified. In this work, the CO_2_ adsorption sites and their interaction within the MOF were identified using a combined *in situ* Fourier transform infrared (FTIR) spectroscopy, density functional theory (DFT) calculations, and Grand Canonical Monte Carlo (GCMC) simulations. CO_2_ molecules were found to reside in the MOF pore through multiple interactions enhancing CO_2_ adsorption at particularly low concentrations. Notably, ZnDTZ showed exceptionally fast CO_2_ adsorption kinetics, achieving 90% of its equilibrium capacity within 191 s compared to 355 s observed on CALF-20 under similar conditions (303 K, 5% CO_2_/N_2_). DFT and GCMC analyses indicate that this remarkably fast CO_2_ adsorption is due to barrierless diffusion of CO_2_ molecules within the pore with homogeneously distributed pore potential of ZnDTZ, a finding corroborated by experimental analysis of the adsorption kinetics.

## 2 Experimental Section

### 2.1 Synthesis of ZnDTZ Powder

Zinc carbonate basic (Zn, 11.38 g), oxalic acid dihydrate (Ox, 6.36 g), and 3,5-diamino-1,2,4-triazole (DTZ, 10.00 g) were dissolved in 300.0 mL of DI water and stirred to form a cloudy white suspension. The resulting mixture was transferred into a media bottle, and a hydrothermal reaction was carried out at 363 K for 6 h. Afterward, the reaction mixture was allowed to cool to room temperature before removal from the oven. The product was collected by centrifugation at 7000 rpm for 5 min, washed with methanol (3 times/day), and then dried overnight in an oven at 80 °C. The yield was ca. 70% based on the amount of zinc salt used. Observed CHN % values for [Zn_2_(C_2_O_4_)­(C_2_H_4_N_5_)_2_]·2H_2_O (calculated values): *C* = 15.36 (15.98); *H* = 2.69 (2.68); *N* = 31.18 (31.05).

### 2.2 Characterizations

Powder X-ray diffraction (PXRD) data were collected on a Bruker D8 Advance using Cu Kα radiation (λ = 1.5406 Å). The patterns were collected across 2θ ranges of 5–50° with a scan rate of 0.2° s^–1^ at 0.02°. The morphology of the samples was examined by scanning electron microscopy (SEM) on a Focused Ion beam field emission scanning electron microscope (FEI, model Versa) (FIB FE-SEM) operated at 5 kV. Each sample was placed on carbon tape adhered to a stub. The particle size distribution was processed by ImageJ software.

Thermogravimetric analysis (TGA) of the samples was performed on a Mettler Toledo TGA/DSC 3+ under N_2_ or air gas with a flow rate of 30 sccm. The samples were heated in the temperature range of 25–800 °C at the heating rate of 5 K/min. CHN analysis of the samples was determined by a Leco instrument CHN628 series.

CO_2_ physisorption analysis was conducted using a Micromeritics 3Flex instrument at 273 K. The samples were degassed at 423 K with a heating rate of 5 K/min under vacuum for 12 h before conducting the gas isotherms, and data were processed with 3Flex version 6.03 software. The specific surface area was determined *via* the Brunauer–Emmett–Teller (BET) method using adsorption data from the experiment without data extrapolation at a relative pressure (*P*/*P*_0_) lower than 0.0015. The pore volumes were estimated by the BET method from the desorption branch.

### 2.3 *In Situ* DRIFTS Measurements

*In situ* diffuse reflectance Fourier transform infrared spectroscopy (DRIFTS) of CO_2_ adsorption and desorption was performed on a Nicolet iS50 equipped with a mercury–cadmium–telluride detector. The sample was loaded into the cup of a reaction cell (Harrick Scientific) equipped with ZnSe windows. The inlet of the cell was connected to the gas mixing unit, where the flow rate was controlled by mass flow controllers. These mass flow controllers were calibrated by an ADM flowmeter (Agilent). The total flow rate used in the measurement was 30 sccm. The temperature of the reaction cell was controlled by a combination of a water circulator and a heater. The sample (70 mg) was first pretreated by heating the sample in the reaction cell at 423 K for 3 h under N_2_ flow (99.999%) to remove physisorbed water molecules. The sample was subsequently cooled down to 298 K under N_2_. The CO_2_ gas was then flowed to the cell with increasing concentrations of 3, 5, 10, and 15% balanced with N_2_. The spectra were recorded in the range of 650–4000 cm^–1^ with a resolution of 4 cm^–1^ and 64 scans using KBr as a background.

### 2.4 Computational Details

#### 2.4.1 Density Functional Theory (DFT)

The mechanistic properties at the quantum mechanics level were explored within the framework of density functional theory (DFT). Due to the high complexity of the potential energy surface within the cavity, binding sites were initially identified using the minima hopping method. Preliminary DFT calculations were performed using grid-based DFT, as implemented in the GPAW package. The projector augmented wave (PAW) method, combined with the vdW–DF2 functional, was employed to account for core–valence electron interactions and long-range nonlocal interactions. The real space grid was sampled with a 0.2 Å interval, while the reciprocal space sampling was done only at the Γ point. At this stage, the local minima were explored with the framework fixed to the experimental structure. All configurations identified during the search were subsequently optimized using more accurate DFT calculations, implemented in the Quantum Espresso package. Fine optimization was performed using the PAW method combined with the PBE exchange-correlation functional. Dispersion interactions were incorporated using the DFT–D3 method. The reciprocal space was sampled with a 3 × 3 × 3 *k*–point mesh, and the energy cutoffs for the wave function and charge density were set to 50 and 400 Ry, respectively. In this stage, both the guest molecules and the framework were relaxed, while the cell shape was constrained to the experimental values. The vibrational properties were studied within the harmonic approximation, based on the potential energy surface and forces derived from Quantum Espresso under the conditions described above. The dynamical matrix diagonalization was carried out using the Atomic Simulation Environment (ASE) library. The adsorption energy was determined using the following equation

$$E_{\mathrm{ads}}=E\left[\mathrm{MOF}+nCO_{2}\right]-E\left[\mathrm{MOF}\right]-\mathrm{nE}\left[CO_{2}\right]$$

where *E*[MOF + *n*CO_2_] is the energy of complex system, *E*[MOF] and *E*[CO_2_] is the energy of clean framework and isolated gas phase CO_2_ with the number of CO_2_ molecules equal to *n*.

#### 2.4.2 Grand Canonical Monte Carlo (GCMC) Simulation

The adsorption properties, including the adsorption isotherm, were simulated using grand canonical Monte Carlo (GCMC) simulations, as implemented in the RASPA software. The universal force field (UFF) was employed for the framework atoms, while the TIP4P, Gracia-Sánchez, and TraPPE models were used to describe H_2_O, CO_2_ and N_2_ molecules, respectively. To represent the atomic charges of the framework, REPEAT charges were calculated from the electrostatic potential derived at the DFT level. For the pure CO_2_ adsorption isotherm, initialization and sampling were performed with 10,000 and 20,000 cycles, respectively, for each pressure point, ranging from 1 Pa to 10^5^ Pa. For the pure H_2_O adsorption isotherm, initialization and sampling were performed with 30,000 cycles each, for pressures ranging from 1 to 4590 Pa. The saturation pressure of TIP4P water, 4590 Pa, was used in this study. A 3 × 3 × 3 supercell was employed, with a cutoff radius of 12 Å for both nonbonded and electrostatic interactions. The Monte Carlo (MC) movements–translation, rotation, swapping, and reinsertion were considered with equal probability. In the flue gas simulation at a total pressure of 10^5^ Pa, the gas mixture consisted of 10% CO_2_, relative humidity corresponding to a saturation pressure of 4590 Pa, and the remainder filled with N_2_ gas.

### 2.5 Gravimetric CO_2_ Adsorption Analysis

Gravimetric CO_2_ adsorption analysis was conducted on a Mettler Toledo TGA/DSC 3+. CO_2_ (99.995%, SI Technology) and N_2_ (99.999%, SI Technology) cylinders were connected to the instrument directly and to a gas mixing unit equipped with mass flow controllers for preparing CO_2_ at different concentrations balanced with N_2_. For the analysis, 10–20 mg of an adsorbent was loaded into a 100 μL Al pan and gently pressed to form a flat surface. The sample was pretreated at the same temperature as for the physisorption analysis under N_2_ flow for 1–2 h until a flat baseline was obtained with a flow rate of 100 sccm in the case of pure CO_2_ adsorption and 150 sccm in the case of mixed 3, 5, 15% CO_2_/N_2_ adsorption, and cooled down to 298, 303, 308, and 318 K under N_2_ flow. For the CO_2_ adsorption, pure CO_2_ (100 sccm) or mixed 3, 5, 15% CO_2_/N_2_ (150 sccm) was flowed into the chamber for 1 h. After that, CO_2_ was desorbed from the sample by switching the gas feed to N_2_ while the temperature was increased to the pretreatment temperature (5 K/min). Buoyancy correction was executed by measuring the empty pan while other parameters were maintained. More details are given in the Supporting Information.

## 3 Results and Discussion

### 3.1 Synthesis and Characterizations of ZnDTZ

Single crystals of ZnDTZ were synthesized *via* a solvothermal reaction of zinc oxalate dihydrate and 3,5-diamino-1,2,4-triazole (H-DTZ) in a mixture of DMF, water, and a small amount of HCl (Sections S1–S2). Based on single-crystal X-ray diffraction analysis and C H N elemental analysis, the compound ZnDTZ is formulated as [Zn­(DTZ)­(oxalate)_0.5_]·H_2_O. Structural analysis reveals that ZnDTZ crystallizes in the monoclinic space group *P*2/*c* and adopts an *sqc* topology^49^ (Figure 1, crystal data in Tables S1–S2).

**Figure 1:**
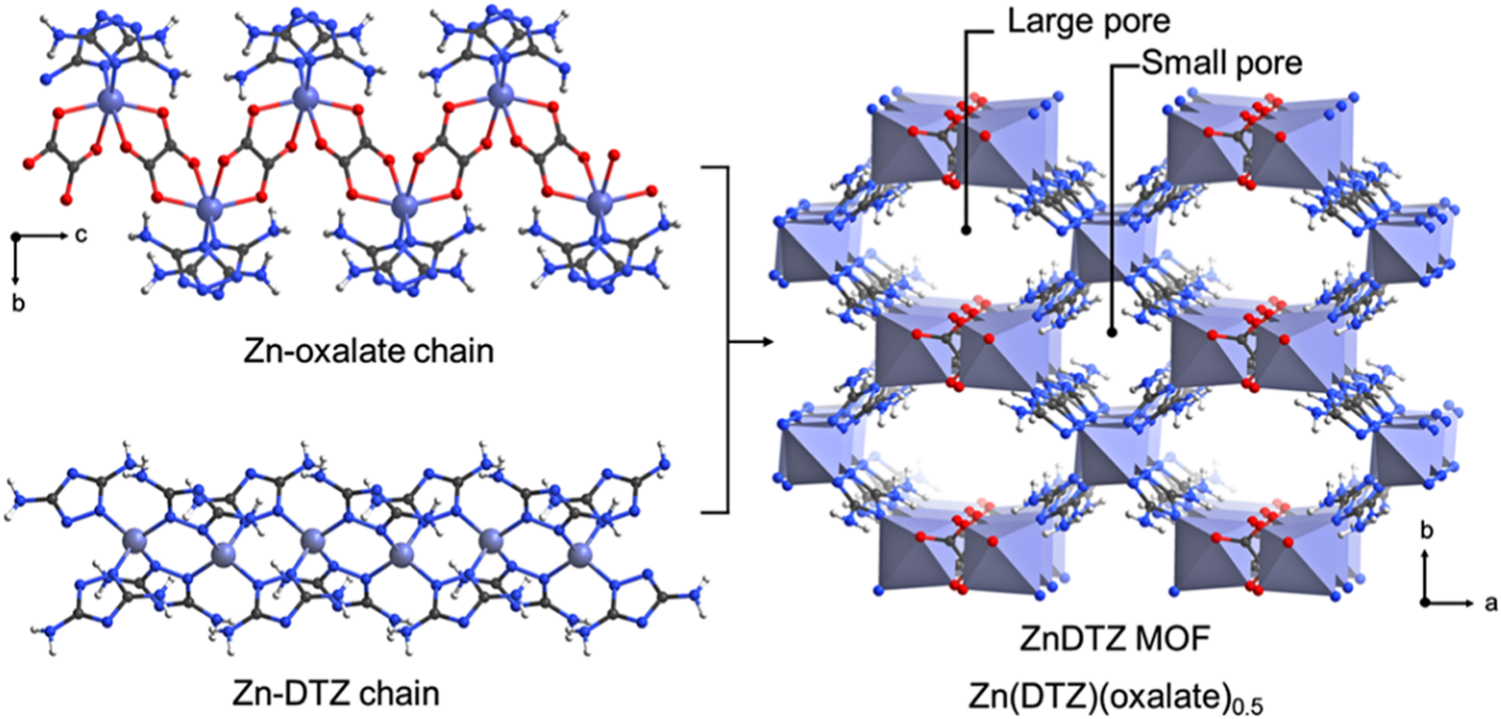
Construction of Zn­(DTZ)­(oxalate)_0.5_ (ZnDTZ) based on the SXRD analysis. Color scheme: Zn, purple or purple polyhedron; C, gray; N, blue; O, red.

The framework consists of two distinct Zn nodes. The Zn1 center exhibits a distorted octahedral geometry (ZnN_2_O_4_), coordinated by two nitrogen atoms from two DTZ ligands and four oxygen atoms of oxalate ligands. These Zn1 centers are bridged through μ_2_–tetradentate oxalate ligands to form one-dimensional (1D) Zn–oxalate chains along the *c*–axis. In contrast, the Zn2 center displays a distorted tetrahedral coordination geometry (ZnN_4_), coordinated by four nitrogen atoms from four DTZ ligands. These Zn2 centers are interconnected together by the N2 and N3 atoms (1– and 2– positions of the triazole ring) of DTZ ligands forming a 1D Zn–DTZ chains also aligned parallel to the *c*–axis. These two types of chains are further linked through the nitrogen atom (N1) of the DTZ ligands at the 4–position of the triazole ring, forming a three-dimensional (3D) framework.

The structure features two distinct 1D open channels orientated along the *c*–axis: one square–shaped (6.07 × 6.09 Å^2^) designated as large pore and one hexagonal (8.39 × 11.99 Å^2^) designated as small pore.

We then optimized the synthesis conditions to produce ZnDTZ under green conditions, allowing the reaction to be carried out in water under ambient pressure, thereby reducing synthesis costs. After screening various solvents, reactant ratios, temperatures, reaction concentrations, and reaction times, we found that ZnDTZ could be synthesized in water at temperatures as low as 363 K under ambient pressure, eliminating the need for expensive high-pressure vessels. Notably, ZnDTZ can be synthesized with a high space–time–yield of 442 kg m^–3^day^–1^ while maintaining phase purity, as evidenced by the close agreement between the powder X-ray diffraction (PXRD) patterns of the ZnDTZ synthesized as single crystals and in powder form (Figures 2a and S1). The pattern was found to closely resemble the pattern reported recently and the comparison of structural information is tabulated in Tables S3–S5.^47,48^

**Figure 2:**
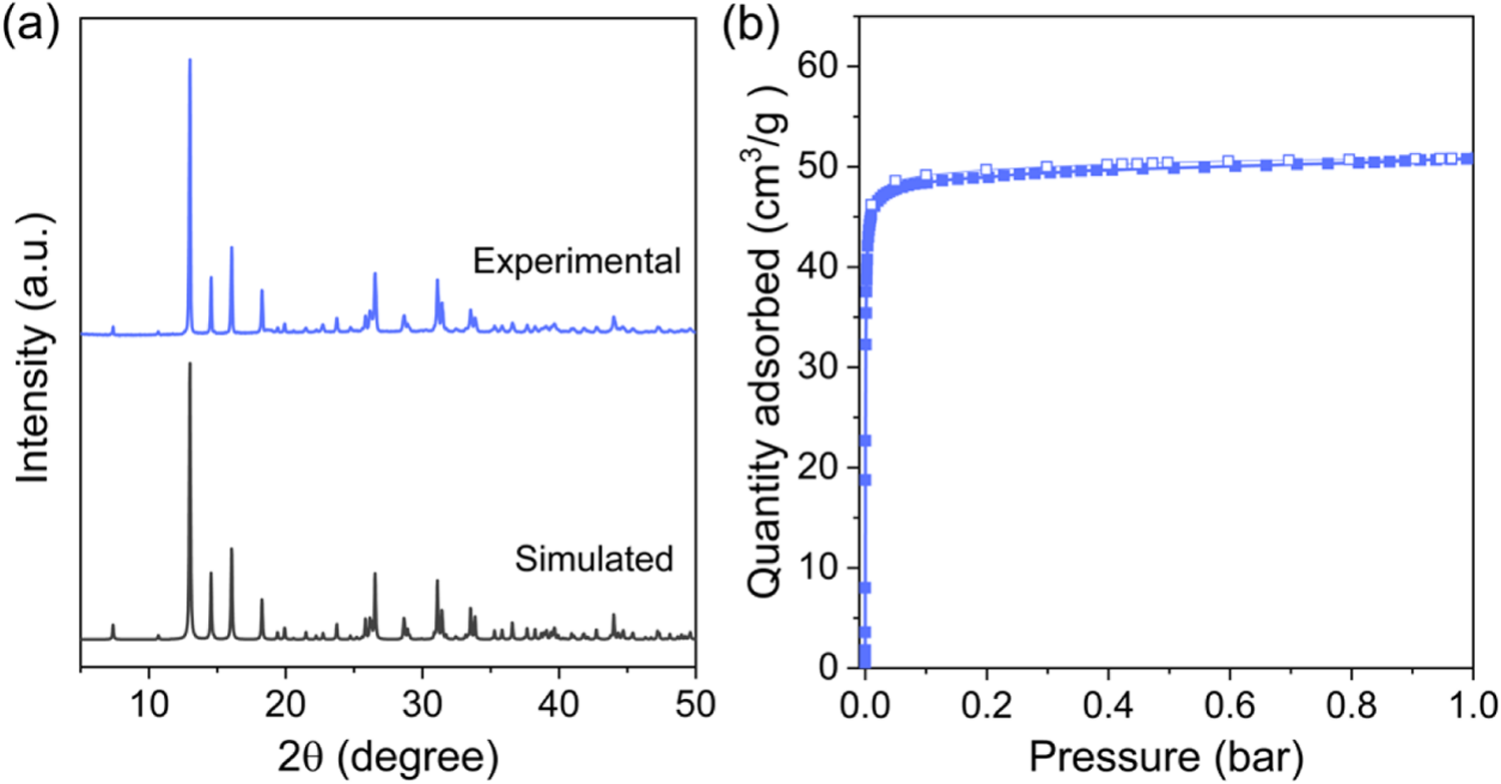
(a) PXRD patterns of as-synthesized and simulated ZnDTZ powder and (b) CO_2_ sorption isotherm at 273 K of ZnDTZ powder, solid and hollow symbols represent adsorption and desorption branches, respectively.

Analysis of the material porosity was initially performed using the N_2_ adsorption isotherm at 77 K. However, due to the small pore size complicating the analysis using N_2_ as the adsorbate, we instead determined the specific BET surface area and total pore volume using the CO_2_ adsorption–desorption isotherm at 273 K (Figure 2b). ZnDTZ exhibited a reversible Type I­(a) isotherm, characteristic of microporous solids with relatively small external surfaces and micropores smaller than 1 nm.^50^ The BET-calculated specific surface area is 240 m^2^/g with a total pore volume of 0.11 cm^3^/g (Figure S2). Scanning electron microscopy (SEM) analysis of the powder showed a particle size of around 10 ± 2 μm (Figure S3).

We then investigated the stability of this MOF in terms of both chemical and thermal stability. First, the MOF was immersed in water with pH values ranging from 1 to 14 at room temperature for 24 h. The MOF showed exceptional stability in water from pH 1 to 12 (Figure S4a) and exhibited excellent hydrothermal tolerance, remaining stable in boiling water for at least 72 h (Figure S4b). Additionally, the MOF is stable in a wide variety of solvents, including hexanes, acetone, DMF, isopropanol, ethanol, methanol, DMA, and acetonitrile (Figure S4c). Under these conditions, no changes in the PXRD patterns were observed, confirming its high stability. Thermogravimetric analysis (TGA) in air and N_2_ showed that the MOF started to lose weight at around 573 K, which is sufficient for most CO_2_ capture applications (Figure S4d).

### 3.2 Single Component Gas Adsorption Isotherm Analysis

Adsorption isotherms are essential for investigating the interaction between gaseous molecules and adsorbents. Single-component adsorption isotherms of CO_2_, N_2_, and H_2_O on the ZnDTZ adsorbent at different temperatures are presented in Figure 3. The CO_2_ adsorption isotherms reveal notable CO_2_ uptake at very low pressures (Figure 3a). At 298 K, the CO_2_ capacities at 0.03, 0.05, and 0.15 bar are 2.13, 2.22, and 2.36 mmol/g, respectively. At 303 K, these values decrease to 1.89, 1.97, and 2.09 mmol/g, and at 308 K, they decline further to 1.49, 1.62, and 1.82 mmol/g. At the highest temperature tested, 318 K, the uptakes are 1.40, 1.53, and 1.73 mmol/g at the same respective pressures (uptake capacities at other temperatures are summarized in Table S9). A comparison of CO_2_ adsorption performance with other MOF materials is provided in Table S6, indicating that ZnDTZ demonstrates promising CO_2_ capture properties at a low CO_2_ partial pressure of around 5% CO_2_ and 1 bar.

**Figure 3:**
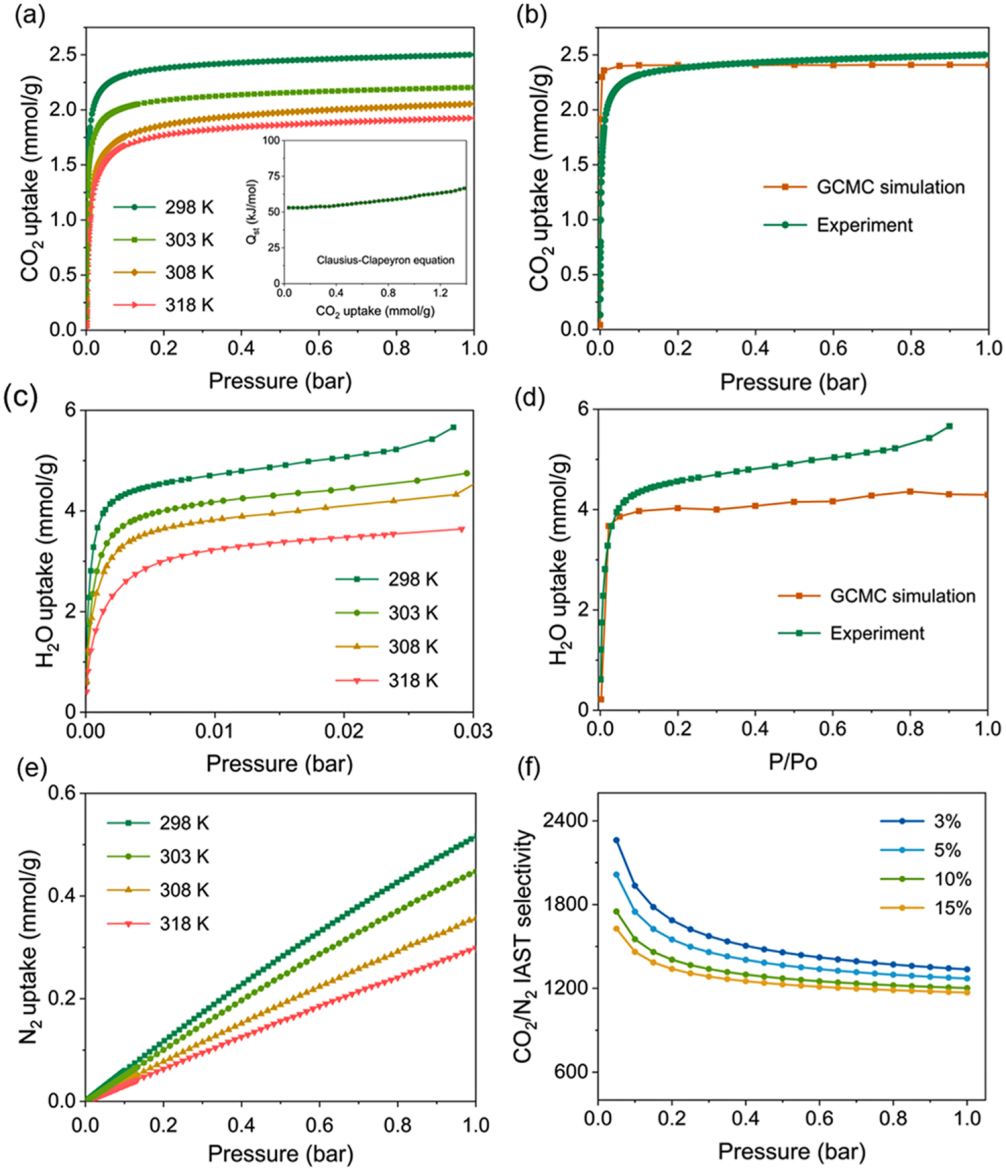
(a) CO_2_ isotherms of ZnDTZ at 298–318 K and *Q*_st_ shown in the inset, (b) CO_2_ isotherms of ZnDTZ at 298 K compared with GCMC simulation, (c) H_2_O adsorption isotherms of ZnDTZ at 298–318 K, (d) H_2_O adsorption isotherms of ZnDTZ at 298 K compared with GCMC simulation of the water isotherm, (e) N_2_ adsorption isotherms at 298–318 K, and (f) CO_2_/N_2_ selectivity of ZnDTZ calculated using the IAST model at 303 K with different feed compositions.

To calculate the isosteric heat of adsorption (*Q*_st_), the Clausius–Clapeyron equation was applied to the CO_2_ adsorption isotherms at 298–318 K (eq S1). At zero CO_2_ loading, the *Q*_st_ was determined to be −50.4 kJ/mol (Figure 3a), placing it at the upper range for physisorption processes.^33^ GCMC simulations of the CO_2_ adsorption isotherm at 298 K were conducted to understand the adsorption behavior (Figure 3b). The simulation shows a Type-I isotherm with the CO_2_ capacity at 1 bar of 2.41 mmol/g, in excellent agreement with the experimental results. Additionally, the *Q*_st_ values of −51.0 kJ/mol obtained from classical force field parametrization and −52.1 kJ/mol from DFT simulation are both closely aligned with the experimental data, validating the accuracy of the simulation parameters and the reliability of the simulation conditions.

For water adsorption, ZnDTZ shows a rather sharp water uptake at a low pressure of 0.005 bar, consistent with the results from GCMC simulation (Figure 3c,d). At higher temperatures, the water capacity decreased, and the inflection point shifted to higher partial pressure. Water contact angle measurement also indicated that the external surface of ZnDTZ particles is hydrophilic (Figure S5). Combined with the water adsorption behavior, this indicates that both external surface and internal pore of ZnDTZ are hydrophilic, making the material suitable for capturing CO_2_ with limited moisture content.^51–53^ Moreover, we measured N_2_ adsorption isotherms at the same temperatures, as N_2_ is a common contaminant in flue gas. At 1 bar, ZnDTZ showed limited uptake of N_2_: 0.51, 0.44, 0.36, and 0.30 mmol/g at 298, 303, 308, and 318 K, respectively (Figure 3e). Based on this data, IAST calculations were conducted using single-component isotherms of CO_2_ and N_2_ at 298, 303, 308, and 318 K (Sections S4 and S8, Figure S6). The CO_2_/N_2_ selectivity (Figure 3f) was calculated for pressures ranging from 0 to1 bar, with the CO_2_ concentration fixed at 3, 5, and 15%. The plot shows that CO_2_ selectivity over N_2_ is maximized at very low pressures, followed by a significant decline occurring between 0 and 0.2 bar, followed by a gradual decrease with increasing pressure. At 303 K and 1 bar, the CO_2_/N_2_ selectivity values are 1336, 1270, 1201, and 1170 for CO_2_ concentrations in N_2_ of 3, 5, 10 and 15%, respectively, with these values decreasing as the temperature increase.

### 3.3 Determination of CO_2_ Adsorption Sites

To investigate the adsorption behavior of CO_2_ in the MOF, we conducted *in situ* diffuse reflectance infrared Fourier transform spectroscopy (DRIFTS) analysis during both the CO_2_ adsorption and desorption processes. The sample was pretreated under a flow of N_2_ at 423 K for 3 h to remove volatile adsorbates. In the N–H stretching region, peaks were observed at 3475, 3456, 3381, and 3341 cm^–1^, corresponding to the asymmetric and symmetric stretches of the N–H bonds, respectively (Figure 4a).^54,55^ During CO_2_ adsorption, with CO_2_ concentrations ranging from 3 to 15% balanced with N_2_, the asymmetric N–H stretches (3475 and 3456 cm^–1^) merged into a broad peak centered at 3466 cm^–1^. Meanwhile, the symmetric stretch at 3341 cm^–1^ was blue-shifted to 3351 cm^–1^, indicating that the NH_2_ functionalities of the DTZ interacted with CO_2_ molecules.

**Figure 4:**
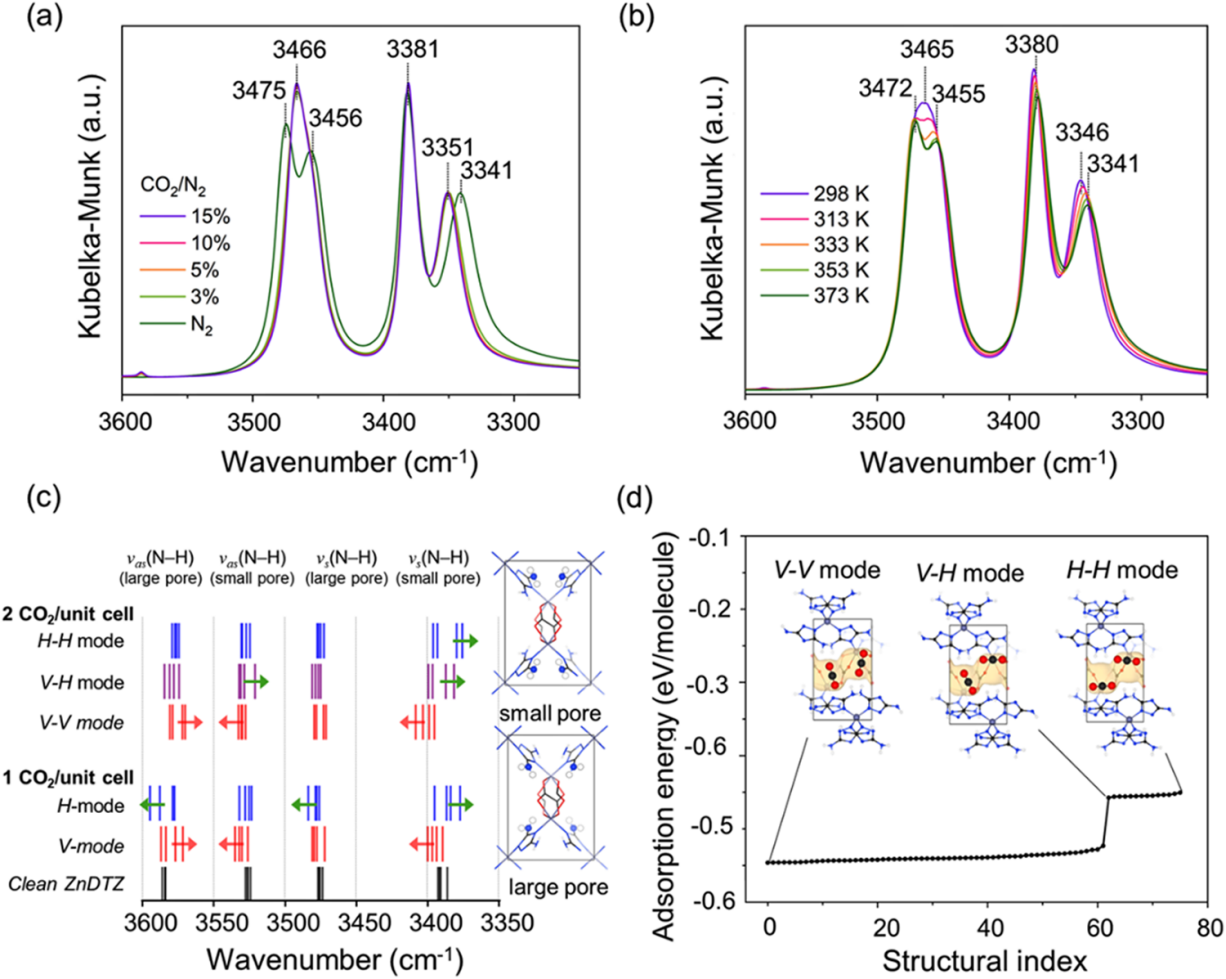
*In situ* DRIFTS spectra of ZnDTZ under varying CO_2_ concentrations in the range of 3600–3250 cm^–1^ during (a) CO_2_ adsorption and (b) CO_2_ desorption. (c) DFT vibrational frequencies of the symmetric (ν_s_) and asymmetric (ν_as_) N–H stretch modes in −NH_2_ for pristine ZnDTZ, and ZnDTZ with one CO_2_ molecule per unit cell, and ZnDTZ with two CO_2_ molecules per unit cell. (d) The stable binding configurations within the channel were obtained from DFT optimization using the minima hopping method for identifying local minima. At high CO_2_ concentrations, the configurations from GCMC simulations closely resemble the V–V mode structure, which was identified as the most stable configuration from DFT, demonstrating agreement between the DFT optimization and GCMC simulations.

The MOF sample was then flushed with N_2_, and the temperature was increased to 313, 333, 353, and 373 K. A progressive change in the N–H stretch peaks was observed as the surface coverage of CO_2_ decreased. The broad asymmetric N–H stretch found at 3465 cm^–1^ split back into two peaks at 3472 and 3455 cm^–1^ (Figure 4b), while the symmetric N–H stretch gradually shifted from 3346 to 3341 cm^–1^.

The specific binding configuration was investigated within the DFT framework. Notably, CO_2_ molecules accessed only the large pores of the framework, while the small pores remained unoccupied. At low concentrations, the most stable binding configuration was identified as the vertical (V) mode (Figure S8), where CO_2_ aligns perpendicular to the channel direction, with an adsorption energy of −52.1 kJ/mol per molecule. In contrast, the horizontal (H) mode, where the CO_2_ aligns parallel to the channel direction, showed slightly weaker binding with an adsorption energy of −40.5 kJ/mol.

The radial distribution function (RDF) analysis revealed the multisite binding characteristic, with the −NH_2_ groups of the MOF interacting with the oxygen atoms of CO_2_ at distances centered at 2.33 and 2.91 Å, and the oxalate oxygen atoms interacting with the carbon atoms of CO_2_ at 2.82 Å (Figure S10a). Furthermore, this interaction was confirmed by noncovalent interaction analysis based on the density overlap regions indicator (DORI) method,^56^ as shown in Figure S10c.

At higher CO_2_ concentrations, where the adsorption capacity reaches saturation (Figure 4c), the CO_2_ molecules are packed in an ordered fashion at neighboring sites, forming a vertical packing configuration within the one-dimensional channel (V–V mode). To correlate the theoretically predicted stable binding mode with experimental observations, the vibrational frequencies of the N–H of the framework and the C–O stretches of CO_2_ were evaluated at both low and high CO_2_ concentration conditions using DFT. As illustrated in Figure 4d, considering the symmetry of the framework, the −NH_2_ groups can be classified into two unique positions located in different environments, leading to the splitting of both asymmetric (ν_as_) and symmetric (ν_s_) N–H stretches. Consequently, a total of four vibrational positions are expected to appear in the N–H stretching region. Based on these calculations, we considered five possible scenarios for packing CO_2_ molecules in the pores, each resulting in distinct shifts of the N–H stretching peaks, allowing us to map the alignment of the CO_2_ molecules in the pores with the experimental data.

As CO_2_ concentration increases, the number of CO_2_ molecules increases from one to two per unit cell, resulting in specific spectral shifts observed in the vibrational modes. Notably, a blue shift in the lowest symmetric stretch occurs, which is found exclusively in the V–V mode. Additionally, a red shift in the higher asymmetric stretch and a blue shift in a lower asymmetric N–H stretches are also observed, but only in the V–V mode. These spectral shifts align closely with those observed in the DRIFTS spectrum in Figure 4a, indicating that the V and V–V configurations are predominant under low and high CO_2_ concentrations, respectively.

The DRIFTS spectra in the CO_2_ stretching region during the adsorption and desorption processes are displayed in Figures S7 and 5, respectively. During CO_2_ adsorption (Figure S7), the peak at 2360 cm^–1^ corresponds to gas-phase CO_2_, while the peaks at 2342 and 2330 cm^–1^ are associated with adsorbed CO_2_, suggesting the presence of two distinct types of adsorbed CO_2_ within the ZnDTZ pores.^57^ During desorption, both peaks at 2344 and 2330 cm^–1^ were present at 298 K and only the peak at 2342 cm^–1^ remained after heating up to 373 K (Figure 5). To gain further insights into the CO_2_ adsorption behavior of ZnDTZ, we compared it with the benchmarking material CALF-20 because of its high adsorption capacity and comparable structural composition providing a basis for the following discussion. For CALF-20, *in situ* DRIFTS during the CO_2_ desorption showed only one peak located at 2341 cm^–1^ (Figure 5b).

**Figure 5:**
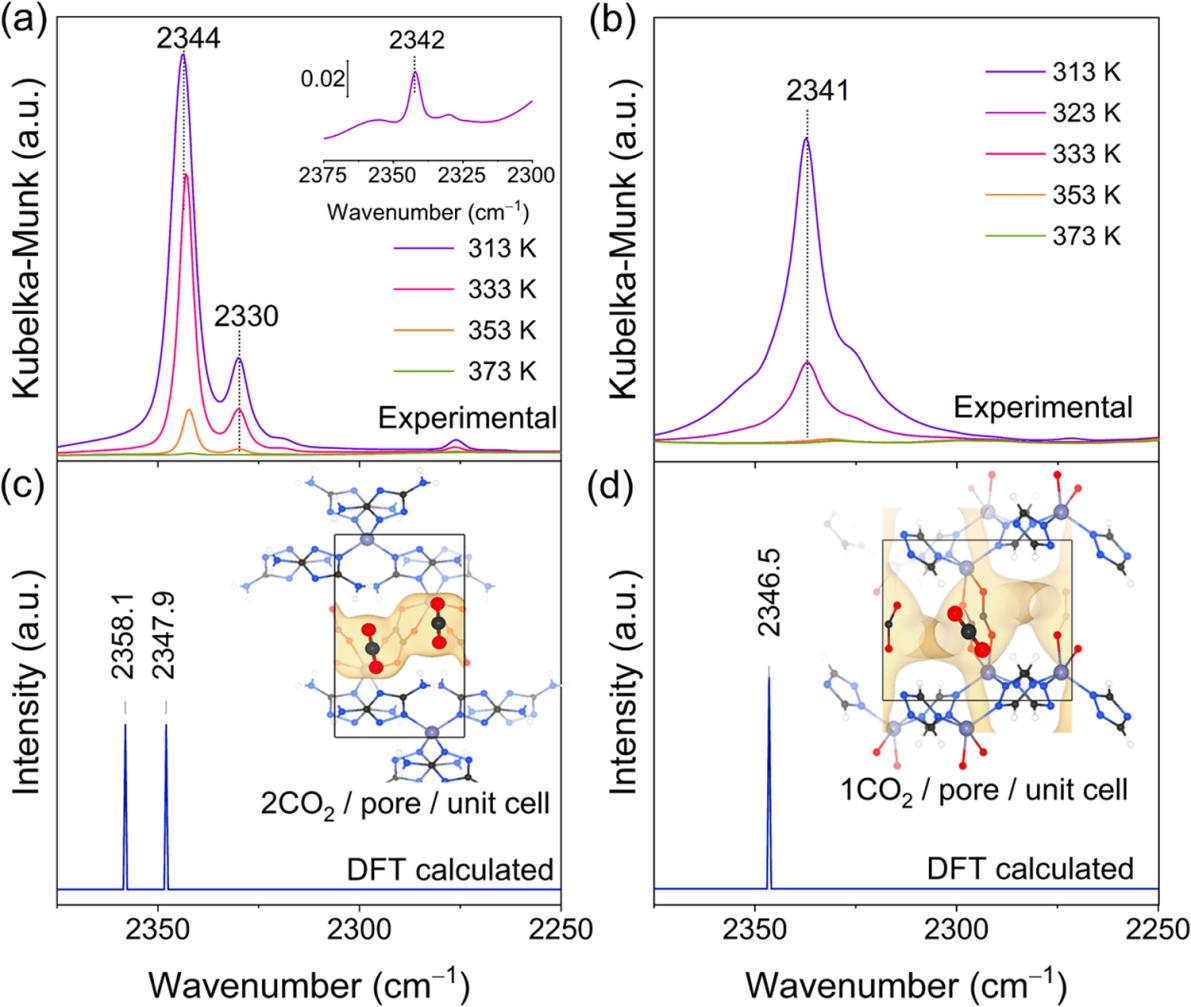
Experimental IR spectra of *in situ* DRIFTS for CO_2_ desorption at different temperatures, in comparison with DFT vibrational frequencies for C–O stretch in (a, c) ZnDTZ and (b, d) CALF-20.

Distinct differences in the vibrational characteristics of CO_2_ in the ZnDTZ and CALF-20 frameworks were revealed through vibrational spectra calculated at the DFT level. For CALF-20 (Figure 5d), a single peak at 2346 cm^–1^, corresponding to the asymmetric stretching of adsorbed CO_2_, was predicted, which is in good agreement with the experimental results (Figure 5b). Notably, for ZnDTZ, the asymmetric stretching mode of adsorbed CO_2_ exhibited splitting into two peaks, separated by 10 cm^–1^. This splitting is attributed to the adsorption of CO_2_ molecules at neighboring sites (Figure 5c) in V–V mode, which is the primary configuration in the GCMC simulations. These findings confirm distinct CO_2_ adsorption behaviors in the two frameworks. Although there are differences between the exact vibrational frequencies in the calculated and experimental values, the trends in frequency shift are consistent. This suggests that CO_2_ is adsorbed as a single molecule per pore in CALF-20, whereas ZnDTZ accommodates two CO_2_ molecules per unit cell, primarily governed by V–V mode interactions, with the – NH_2_ group involved as the noncovalent interaction site.

### 3.4 CO_2_ Gravimetric Analysis

We performed gravimetric analysis of the CO_2_ adsorption–desorption processes to evaluate the material’s CO_2_ uptake and adsorption kinetics under dynamic conditions, simulating the dilute CO_2_ gas typically found in flue gases from various sources. Regarding CO_2_ capacity (Figure 6(a–f), Table S9), ZnDTZ can adsorb CO_2_ up to 1.97 mmol/g at 303 K, 1.93 mmol/g at 308 K, and 1.88 mmol/g at 318 K when exposed to a 15% CO_2_/N_2_ mixture. In comparison, CALF-20 adsorbs CO_2_ up to 2.24 mmol/g at 303 K, 2.06 mmol/g at 308 K, and 1.73 mmol/g at 318 K.

**Figure 6:**
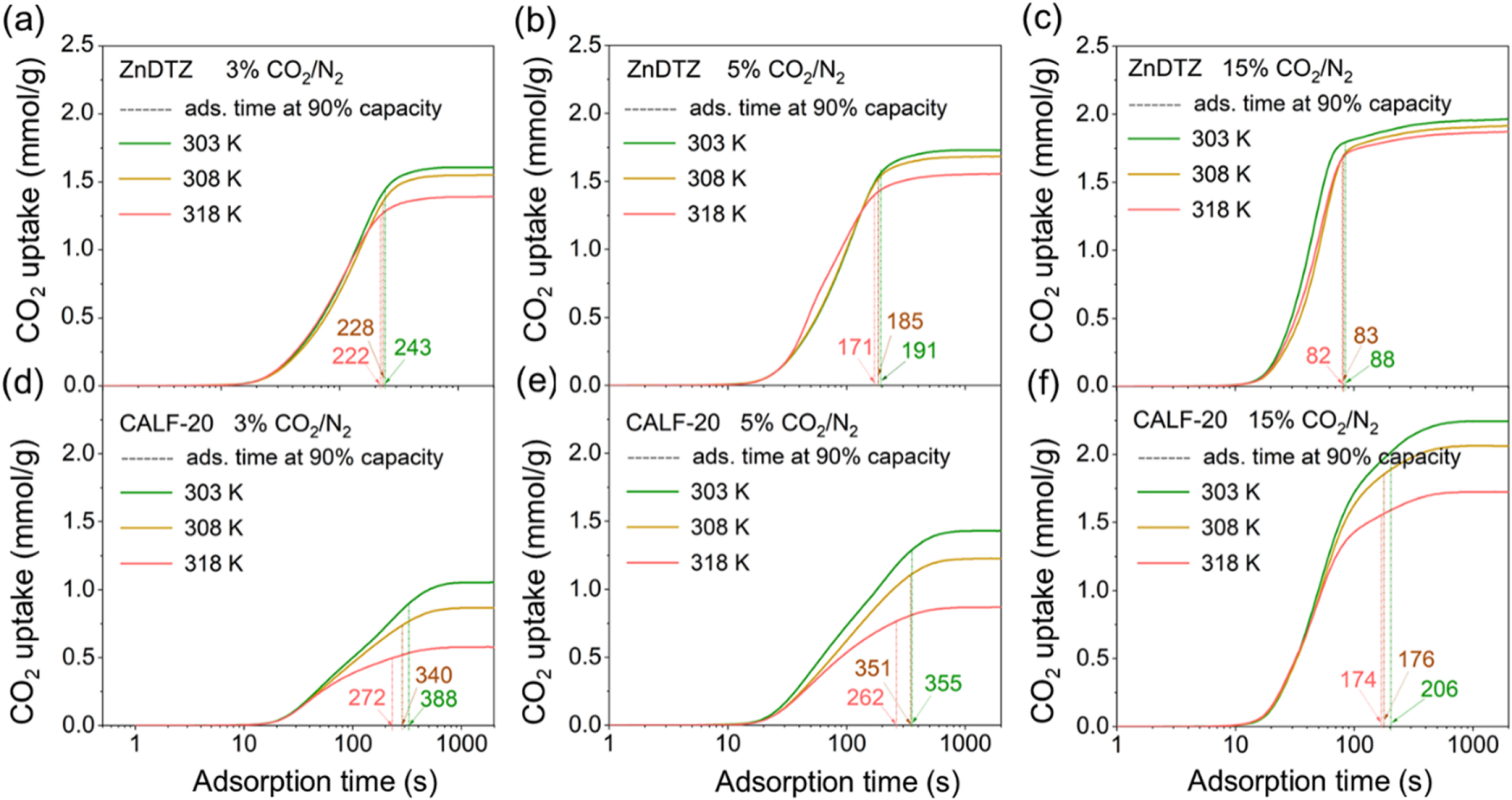
Gravimetric CO_2_ adsorption under 3, 5, and 15% CO_2_/N_2_ at 303, 308, and 318 K of (a–c) ZnDTZ and (d–f) CALF-20. Vertical dashed lines indicate the time required to reach 90% of the CO_2_ equilibrium capacity.

At a lower concentration of 5% CO_2_/N_2_, ZnDTZ adsorbs CO_2_ up to 1.73 mmol/g at 303 K, 1.69 mmol/g at 308 K, and 1.56 mmol/g at 318 K, whereas CALF-20 adsorbs CO_2_ at 1.43 mmol/g at 303 K, 1.23 mmol/g at 308 K, and 0.86 mmol/g at 318 K. At the lowest concentration of 3% CO_2_/N_2_, ZnDTZ’s CO_2_ adsorption capacities are 1.61 mmol/g at 303 K, 1.55 mmol/g at 308 K, and 1.40 mmol/g at 318 K, while CALF-20 adsorbs CO_2_ at 1.06 mmol/g at 303 K, 0.87 mmol/g at 308 K, and 0.58 mmol/g at 318 K. This trend aligns with the data obtained from the CO_2_ adsorption isotherms, indicating that ZnDTZ performs well even at a CO_2_ concentration of 3% CO_2_/N_2_, which can be found in natural gas power plants.

Another key factor determining material performance is the kinetics of CO_2_ adsorption, which directly affects productivity during gas adsorption.^5,58^ This aspect is particularly important for rapid temperature-swing or rapid pressure-swing adsorption, which are proposed methods to enhance the performance of solid adsorbents. To evaluate the CO_2_ adsorption kinetics, we utilized a gravimetric analyzer, which allows for direct tracking of CO_2_ adsorption progress as a function of time. In this experiment, the sample was pretreated under N_2_ to remove volatile adsorbates until a stable weight change was reached, after which it was exposed to a CO_2_/N_2_ mixture at CO_2_ concentrations of 3, 5, and 15%, balanced with N_2_ at temperatures of 303, 308, and 318 K. The results, summarized in Table S10, indicate that ZnDTZ achieved 90% of its equilibrium CO_2_ adsorption capacity significantly faster (3.18 min) than CALF-20 (5.92 min) at 303 K and 5% CO_2_/N_2_. Notably, this difference in adsorption kinetics is not attributable to particle size since the CALF-20 used in this work had a particle size of 0.5 μm ± 0.1 nm which is even smaller than that of ZnDTZ. The CO_2_ diffusion coefficients of ZnDTZ were also estimated and compared with those other reported materials, indicating that ZnDTZ exhibits a superior CO_2_ diffusivity (Tables S11 and S12).

To further investigate the CO_2_ adsorption kinetics, we fitted the adsorption curves obtained from gravimetric analysis to commonly used models, including the pseudo-first order and pseudo-second order models. The sum square errors (SSE) were employed to determine suitability of the model fitting. This analysis helps elucidate the underlying adsorption mechanisms.^59–61^ At a concentration of 15% CO_2_/N_2_ across all tested temperatures (303–318 K), the CO_2_ adsorption kinetics for ZnDTZ were best described by pseudo-first order model, while CALF-20 exhibited a profile consistent with the pseudo-second order model (Figures S14–S15 and Table S13).^62^ The pseudo-first order model is based on the assumption that the adsorption rate is proportionally correlated to the number of vacant adsorption sites.^63^ In contrast, the pseudo-second model assumes an interaction between the adsorbate and the adsorbent. These findings indicate that the CO_2_ adsorption process in ZnDTZ is reversible, a characteristic commonly found in physisorption processes.^64–66^

To gain deeper insight into the kinetic adsorption of CO_2_ in ZnDTZ and CALF-20, a nudged elastic band (NEB) analysis was performed at the DFT level (details of the methodology are provided in Section S10). Notably, the nearly barrierless character (7.10 kJ/mol) of CO_2_ hopping between neighboring sites in ZnDTZ was confirmed (Figure 7a), suggesting that the migration process is facile. In contrast, the interpore migration barriers of CO_2_ in CALF-20 were estimated to be 20.4 and 34.7 kJ/mol for two possible channels (Figure 7b). These higher energy barriers indicate that CO_2_ diffusion kinetics in CALF-20 are partially hindered by stronger host–guest interactions compared to ZnDTZ.

**Figure 7:**
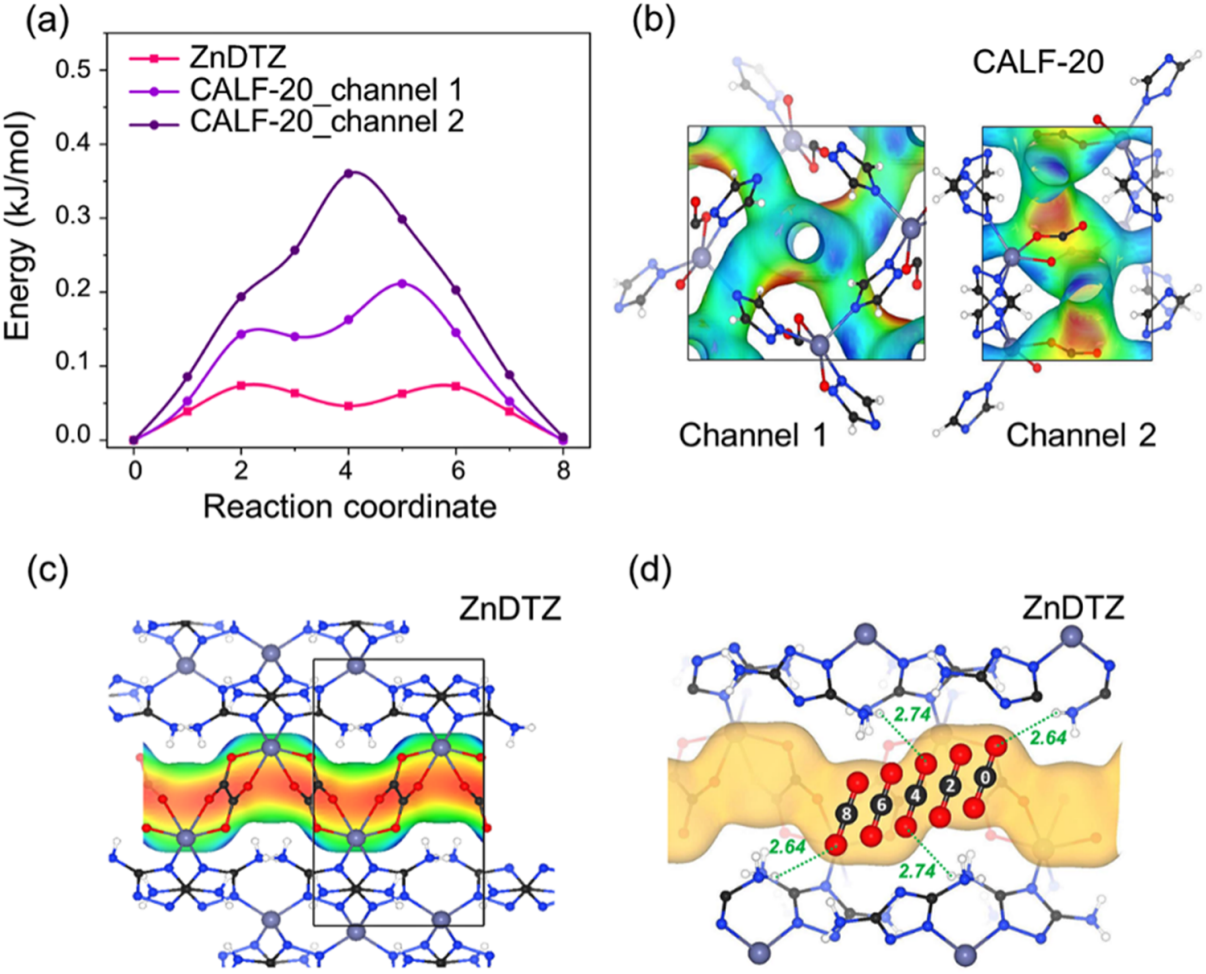
(a) Nudged elastic band (NEB) analysis within the DFT framework for CO_2_ hopping between neighboring local minima of ZnDTZ and CALF-20, (b) Electrostatic potential distribution in CALF-20 for CO_2_ migration through channel 1 and channel 2, (c) electrostatic potential projected onto the one-dimensional pore wall of ZnDTZ highlighting its equipotential characteristics, with red and blue colors representing negative and positive electrostatic potentials, respectively. (d) Migration images of CO_2_ along the pore of ZnDTZ corresponding to the barrier in (a).

Interestingly, at low CO_2_ concentrations, the rapid diffusion kinetics within the one-dimensional channel of ZnDTZ, despite moderately strong binding, is somewhat counterintuitive. This unique characteristic is attributed to the equipotential profile in the electrostatic potential along the channel walls as illustrated in Figure 7c–d. The negative regions align with oxalate oxygens, while the positive regions are distributed along the −NH_2_ hydrogens. This arrangement creates a contiguous adsorption site with a relatively uniform electrostatic field, facilitating the low–energy translation of CO_2_ molecules. The average adsorption energy, calculated over the GCMC trajectory, is −51 kJ/mol, with electrostatic interactions contributing 36% of this energy, while the remainder arises from van der Waals interactions. In comparison, the electrostatic contribution in CALF-20 is only 20% of an adsorption energy of −33 kJ/mol. Thus, the coexistence of multiple binding sites for CO_2_ in ZnDTZ combined with the unique one-dimensional electrostatic potential along the channel wall, is responsible for the experimentally observed rapid CO_2_ adsorption kinetics.

With regards to the CO_2_ desorption process, ZnDTZ desorbs at 374 K while CALF-20 desorbs at a lower temperature of 332 K (Figure S16). This difference can be attributed to the stronger interaction of CO_2_ with the ZnDTZ framework. In terms of cyclability, solid adsorbents with reliable regenerability can significantly reduce the cost of CO_2_ capture in practical applications. Therefore, we tested the cycling stability of ZnDTZ using a CO_2_ concentration of 5% balanced with N_2_ at an adsorption temperature of 303 K for 10 min. For the desorption phase, the temperature was raised from 303 to 423 K at a rate of 10 K/min under pure N_2_ without a holding time. After 200 cycles, ZnDTZ retained 100% of its initial CO_2_ capacity of 1.73 mmol/g (Figure S17). Moreover, the spent ZnDTZ after the cycling test was analyzed using PXRD, comparing the results with those of fresh ZnDTZ (Figure S18). The PXRD patterns of both the spent and fresh samples were similar, suggesting that the structure of ZnDTZ remains stable under these conditions.

### 3.5 CO_2_ Adsorption under Humid Conditions

Competitive CO_2_/N_2_ adsorption under dry and humid conditions at 303 K was carried out using the dynamic column breakthrough technique (see experimental details in Section S6). Before each experiment, the sample in the column was pretreated at 423 K for 3 h, under N_2_, and the effects of water on the CO_2_ uptake were analyzed under three scenarios (Figure S19). Under completely dry conditions where MOF was exposed to dry 5% CO_2_/N_2_ at 303 K, ZnDTZ exhibited a CO_2_ adsorption capacity of 1.64 mmol/g. In the second scenario, ZnDTZ was exposed to humidified 5% CO_2_/N_2_ at 40% relative humidity (RH) at 303 K, the CO_2_ adsorption capacity significantly decreased to 0.80 mmol/g. Under fully wet conditions where MOF was initially preconditioned in 40% RH under N_2_ at 303 K followed by the CO_2_ adsorption at 5% CO_2_/N_2_ at 40% RH and 303 K, the CO_2_ uptake was severely impacted and decreased to 0.01 mmol/g. These results strongly suggest that optimal CO_2_ adsorption performance of ZnDTZ depends on the initial removal of water vapor from the gas stream.

## 4 Conclusions

In this work, ZnDTZ was synthesized through a solvothermal reaction for single crystals and a scalable aqueous reaction under ambient pressure for the powder form. The material was thoroughly characterized to confirm its physical properties and stability using PXRD, SEM, TGA, and CO_2_ adsorption analysis. ZnDTZ exhibited promising CO_2_ adsorption capabilities at 0.05 bar of CO_2_ with an uptake of 1.97 mmol/g at 303 K, demonstrating its capability for low-concentration CO_2_ adsorption. In-depth investigations using combined *in situ* DRIFTS, and GCMC coupled with DFT calculations revealed the interaction between CO_2_ and the MOF, providing insights into the distinct binding sites and configurations of CO_2_ within the large pores of ZnDTZ. The analysis of CO_2_ adsorption kinetics indicated that ZnDTZ achieved ultrafast CO_2_ uptake following a pseudo-first order kinetic model. DFT calculations attribute the rapid CO_2_ adsorption in ZnDTZ to its unique arrangement of adsorption sites producing a quasi-equipotential profile along the channel walls. These findings underscore the potential of ZnDTZ as a highly efficient solid adsorbent for CO_2_ capture, particularly for diluted gas streams. Furthermore, this work highlights the importance of rationally designing MOF structures to achieve both high adsorption capacity and rapid kinetics, offering valuable insights into the design of pore surfaces and laying the groundwork for developing next-generation adsorbents for carbon capture applications.

## Supplementary Material



## Notes

Detailed synthesis of ZnDTZ, characterization methods and data including SXRD, PXRD, CO_2_/N_2_ selectivity, gravimetric gas adsorption analysis, dynamic breakthrough measurements, *in situ* DRIFTS, computational simulations, and kinetic fitting data (PDF)
