# Supplementary material for: Ultrafast CO2 Capture from Dilute Streams in Quasi-Equipotential Pores of Metal–Organic Frameworks
Source: ACS Appl Mater Interfaces. 2025 Jul 9;17(29):41911–22. doi: 10.1021/acsami.5c05994 (PMC12291079; doi:10.1021/acsami.5c05994)
Supplement: Supplementary file 1 [file am5c05994_si_001.pdf]

Supporting Information

Ultrafast CO_2 Capture from Dilute Streams in Quasi-Equipotential Pores of Metal–Organic Frameworks

Siriporn Kosawatthanakun,[a] Poobodin Mano,[a] Pawan Boonyoung,[a] Nadhita Chanchaona,[b] Kittipong Chainok,[c] Suwadee Jiajaroen,[c] Supaporn Nualyai,[a] Kajornsak Faungnawakij,[a] Supawadee Namuangruk,[a] and Bunyarat Rungtaweevoranit [a*]*

[a] National Nanotechnology Center (NANOTEC), National Science and Technology Development Agency (NSTDA), Pathum Thani 12120, Thailand

[b] National Metal and Materials Technology Center (MTEC), National Science and Technology Development Agency (NSTDA), Pathum Thani, 12120, Thailand

[c] Thammasat University Research Unit in Multifunctional Crystalline Materials and Applications (TU-MCMA), Faculty of Science and Technology, Thammasat University, Pathum Thani, 12121, Thailand

Corresponding Authors

*E-mail: bunyarat.run@nanotec.or.th; supawadee@nanotec.or.th

Section S1. Chemical material and synthesis procedures

Chemicals and Materials

All reagents and solvents were used as received without any further purification: zinc oxalate dihydrate ($ZnC_2O_4 \cdot 2H_2O$, 99%, Sigma Aldrich), 3,5-diamino-1,2,4-triazole, (DTZ, $C_2H_5N_5$, 98%, TCI), zinc carbonate basic ($ZnCO_3$, >58% Zn, Sigma-Aldrich), oxalic acid dihydrate, $(COOH)_2 \cdot 2H_2O$, 99.5%, Univar, Ajax Finechem), hydrochloric acid (HCl, 35%, LOBA CHEMIE), N,N-dimethylformamide AR grade (DMF, 99.9%, RCI Labscan), methanol AR grade (MeOH, 99.9%, RCI Labscan), deionized water (DI).

Synthesis of single crystals of ZnDTZ MOFs

The single crystals were prepared by mixing zinc oxalate dihydrate (0.10 g, 0.52 mmol) and 3,5-diamino-1,2,4-triazole (0.053 g, 0.52 mmol) in a solution containing 3.0 mL of DMF and 3.0 mL of deionized (DI) water. The mixture was stirred to form a cloudy white suspension, to which 100 μL of HCl was added. The resulting suspension was stirred at room temperature for 15 min and subsequently transferred into a 20 mL Teflon-lined autoclave. The solvothermal reaction was carried out at 423 K for 48 h and the autoclave was slowly cooled to room temperature before being removed from the oven. The crystals were thoroughly washed with DMF and methanol (3 times/day each) and dried in air.

Section S2. Single-crystal X-ray diffraction

X-ray diffraction data were acquired using a Bruker D8 QUEST CMOS operating at a temperature of 296(2) K. Data were measured utilizing ω and ϕ scans with Mo-Kα radiation (λ = 0.71073 Å). The total number of runs and images was determined based on the strategy calculation from the APEX5 program,[1] and unit cell indexing was refined utilizing SAINT. Data reduction and scaling were performed using SAINT, and SADABS was used for the absorption correction process. The structure was determined using the ShelXT structure solution, incorporating a combination of Patterson and dual–space recycling methods.[2] The structure was refined using the least squares method with ShelXL.[3] All non-hydrogen atoms were refined anisotropically. The N–H hydrogen atoms were located in different Fourier maps but refined with N–H = 0.85 ± 0.02 Å. The water molecule showed very large displacement parameters and was divided into two fragments with occupancies of 0.47 and 0.53. The hydrogen atoms bonded to the water molecule were refined with a riding model.

Section S3. Single component gas sorption analysis

Manometric adsorption of CO_2, H_2O, N_2 gas was performed using Micromeritics 3Flex instrument. The samples (approximately 100 mg) were degassed at 423 K for 12h. Gas adsorption isotherms were collected at 298, 303, 308, and 318 K. The temperature of the samples during the measurements were controlled by a water bath connected to a water circulating temperature controller. The isosteric heat of adsorption energy (Q_{st}) was calculated from the CO_2 adsorption isotherms via the Clausius-Clapeyron equation (Equation S1)

$$ln\left(\frac{P_1}{P_2}\right) = \frac{-\Delta H}{R}\left(\frac{1}{T_2} - \frac{1}{T_1}\right) \qquad \text{Equation S1}$$

Section S4. CO_2/N_2 selectivity determination

The selectivity of CO_2 over N_2 reflects the relative adsorption of CO_2 molecules compared to N_2 molecules in the adsorbed phase. This selectivity (S_{CO_2/N_2}) can be expressed mathematically as shown in Equation S2:

$$S_{CO_2/N_2} = \left(\frac{q_{CO_2}}{q_{N_2}}\right) / \left(\frac{f_{CO_2}}{f_{N_2}}\right) \qquad \text{Equation S2}$$

where q_{CO_2} and q_{N_2} represent the amounts of CO_2 and N_2 adsorbed (in moles) on the active sites of the ZnDTZ at equilibrium, and f_{CO_2} and f_{N_2} are the fugacities of CO_2 and N_2, respectively, in the gas mixture.[4]

To calculate these parameters, the adsorption isotherm of the CO_2-N_2 mixture is required. Direct measurement of multicomponent isothermal adsorption, however, is often challenging. Therefore, predicted adsorption isotherms are commonly used. The Ideal Adsorption Solution Theory (IAST)[5] is widely employed for predicting multicomponent adsorption isotherms of various adsorbents due to its reliability, simplicity, and practicality as an alternative to direct measurements.[6] In an ideal gas scenario, Raoult's law (

Equation S3) is applied to the Equation S2, resulting in Equation S4

$$f_i = P_i^0 x_i;\ i = CO_2, N_2 \qquad \text{Equation S3}$$

$$S_{CO_2/N_2} = P_{N_2}^0 / P_{CO_2}^0 \qquad \text{Equation S4}$$

where P_i^0 represents the sorption pressure of pure gas i at equilibrium. The P_i^0 value can be determined by assuming that the spreading pressure (π) is constant for each pure gas. This parameter can be approximated using the surface potential (Φ), which correlates to the total molar

amount of adsorbed pure i gas (q_i^0) to the bulk fluid fugacity over the pressure range from the initial state to equilibrium (Equation S5)

$$\Phi = \frac{\pi A}{RT} = \int_0^{P_{CO_2}^0} \frac{q_{CO_2}^0}{f} df = \int_0^{P_{N_2}^0} \frac{q_{N_2}^0}{f} df \qquad \text{Equation S5}$$

where A is the surface area per weight of MOF, R is ideal gas constant, and T is the adsorption temperature.[5] Using this equation, the adsorption isotherms of pure CO_2 and pure N_2, measured experimentally at the same temperature, are plugged into the equation to eventually predicted the selectivity.

Methodology

Due to the limited pressure data points in experimental CO_2 and N_2 adsorption isotherms, converting this experimental data to a mathematical model allows for more pressure points, thus providing more reliable results.

The experimental adsorption isotherms of CO_2 and N_2 were fitted using the single-site Langmuir-Freundlich (SSLF) model[7] (Equation S6) through non-linear regression to determine the empirical parameters: the maximum adsorbed molar of pure gas i on the adsorbent surface ($q_{i,max}^0$), the affinity parameter (k_i), and the Langmuir-Freundlich heterogeneity constant (n_i).

$$q_i^0 = \frac{q_{i,max}^0 k_i P^{n_i}}{1+k_i P^{n_i}}; i = CO_2, N_2 \qquad \text{Equation S6}$$

The non-linear regression was performed using Origin Software. The SSLF model was manually added to the fitting function, with the boundary conditions set as $q_{i,max}^0 > 0$ and $k_i > 0$.

After obtaining the predicted equation for pure gas adsorption, the term q_i^0, as a function of P (The term fugacity usually refers for real gas; the equivalent of fugacity describing for ideal gas is called partial pressure[Atkins'PhysicalChemistry]) is substituted into Equation S5 to solve for P_i^0. Addition inputs, including the molar ratio of gas i in the gas mixture (y_i) and the total pressure (P_t), are required from the practitioner to simulate the isothermal multi–gas adsorption scenarios. The relationships between these parameters are shown in the following equations:

$$P_t y_i = P_i^0 x_i \qquad \text{Equation S7}$$

$$x_{CO_2} + x_{N_2} = 1 \qquad \text{Equation S8}$$

$$y_{CO_2} + y_{N_2} = 1 \qquad \text{Equation S9}$$

Section S5. Gravimetric CO_2 adsorption analysis

The kinetics of CO_2 adsorption was analyzed from the TGA data by fitting with both the pseudo first order (PFO) and pseudo second order (PSO) kinetic models of which the equations are shown in equation S10 and S11 respectively.[8, 9]

$$q_t = q_e(1 - e^{-k_1 t}) \qquad \text{Equation S10}$$

$$q_t = \frac{q_e^2 k_2 t}{1 + q_e k_2 t} \qquad \text{Equation S11}$$

The k_2 value as obtained from the PSO model was then used to calculate the activation energy (E_a) from the Arrhenius equation and plot (Equation S12).

$$\ln k = -\frac{E_a}{RT} + \ln A \qquad \text{Equation S12}$$

Section S6. Dynamic Breakthrough analysis

Breakthrough adsorption experiments were conducted using a custom-designed system coupled with a mass spectrometer (MS) for real-time gas analysis at the reactor outlet. The reactor tube was integrated into the system, and all experimental steps were controlled automatically and continuously using NFED software. A bubbler humidifier was incorporated into the dynamic column breakthrough (DCB) apparatus to generate a humidified gas stream. Multiple mass flow controllers and two bubblers were employed to enable independent control of two gas mixtures, which could be selectively introduced into the adsorption column via a switching valve. Prior to each experiment, 500 mg of ZnDTZ (particle size >750 μm) was loaded into a cylindrical stainless-steel column (diameter: 10.4 mm; length: 20 cm). The adsorbent was pretreated under N_2 (5 sccm) at 363 K for 30 min, followed by heating to 423 K for 3 h at a ramp rate of 10 K/min. For dry adsorption measurements, the column was cooled to 303 K and, at t = 0 min, a gas mixture of 5% CO_2 in N_2 (5 sccm) was introduced and continued until the adsorbent reached saturation. For the dry-wet adsorption condition, the column was similarly cooled to 303 K, and at t = 0 min, a gas mixture comprising 5% CO_2, 40% RH of H_2O, balanced N_2 (5 sccm) was fed into the column until complete saturation of both CO_2 and H_2O was achieved. In the humid (wet) adsorption condition, the column was cooled to 303 K, and at t = 0 min, a humidified gas stream (40% RH of H_2O, balanced N_2, 5 sccm) passed through the column until the H_2O concentration stabilized. Subsequently, the gas mixture (5% CO_2, 40% RH of H_2O, balanced N_2, 5 sccm) was introduced until complete saturation of both CO_2 and H_2O was attained. After adsorption, the column was purged with nitrogen for 10 min to remove non-adsorbed species. To confirm the reliability of the measurements, a bypass test was performed under the same experimental conditions.

Section S7. Characterization results

Table S1 Experimental details.

**Crystal data**	
Chemical formula	$C_3H_4N_5O_2Zn{\cdot}H_2O$
M_r	225.50
Crystal system, space group	Monoclinic, $P2/c$
Temperature (K)	296
a, b, c (Å)	8.5562 (2), 11.9893 (2), 7.3084 (1)
β (°)	104.881 (1)
V (Å^3)	724.57 (2)
Z	4
Radiation type	Mo $K\alpha$
μ (mm^{-1})	3.36
Crystal size (mm)	0.22 × 0.2 × 0.2
**Data collection**	
Diffractometer	BRUKER D8 QUEST CMOS PHOTON II
Absorption correction	Multi-scan
T_{min}, T_{max}	0.644, 0.747
No. of measured, independent and observed [$I > 2\sigma(I)$]. reflections	47681, 3056, 2613
R_{int}	0.048
$(\sin \theta/\lambda)_{max}$ (Å^{-1})	0.795
**Refinement**	
$R[F^2 > 2\sigma(F^2)]$, $wR(F^2)$, S	0.025, 0.065, 1.08
No. of reflections	3056
No. of parameters	147
No. of restraints	22
H-atom treatment	Only H-atom coordinates refined
$\Delta\rho_{max}$, $\Delta\rho_{min}$ (e Å^{-3})	0.48, −0.87

Table S2 Geometric parameters (Å, º).

Zn1−O1	2.0805 (11)	Zn2−N2	1.9732 (11)
Zn1−O2^{ii}	2.2608 (12)	Zn2−N3^{v}	1.9983 (12)
Zn1−N1	2.0591 (12)		
O1−Zn1−O1^{i}	154.86 (6)	N1−Zn1−O2^{iii}	165.64 (5)
O1−Zn1−O2^{ii}	84.38 (5)	N1−Zn1−O2^{ii}	93.03 (5)
O1−Zn1−O2^{iii}	76.13 (5)	N1^{i}−Zn1−N1	97.61 (7)
O2^{iii}−Zn1−O2^{ii}	78.28 (7)	N2−Zn2−N2^{iv}	114.75 (7)
N1−Zn1−O1	91.79 (5)	N2−Zn2−N3^{v}	107.04 (5)
N1−Zn1−O1^{i}	104.80 (5)	N2−Zn2−N3^{vi}	108.98 (5)
N1^{i}−Zn1−O2^{iii}	93.03 (5)	N3^{v}−Zn2−N3^{vi}	110.01 (8)

Symmetry codes: (i) −*x*+1, *y*, −*z*+1/2; (ii) *x*, −*y*+1, *z*+1/2; (iii) −*x*+1, −*y*+1, −*z*.

Table S3 Crystallographic information.

Crystal data	ZnDTZ	NICS-24	IISERP-MOF36
Chemical formula	$C_3H_4N_5O_2Zn\cdot H_2O$	$C_6H_8N_{10}O_4Zn_2$	$C_3H_4N_5O_3Zn$
Mw	225.50	415.0	223.49
Crystal system	Monoclinic	Monoclinic	Monoclinic
space group	*P*2/*c*	$P2_1/c$	*P*2/*c*
Temperature (K)	296	293	150
a (Å)	8.5562 (2)	8.5703 (3)	8.464 (2)
b (Å)	11.9893 (2)	23.9768 (9)	12.025 (3)
c (Å)	7.3084 (1)	7.3145 (3)	7.252 (2)
β (°)	104.881 (1)	104.892 (2)	104.717 (10)
V (Å^3)	724.57 (2)	1452.6 (1)	713.9 (3)
Z	4	12	4
Reference	This work	Klemenčič et al., (2023)[1]	Singh et al., (2025)[2]

Table S4 Comparison of selected interatomic distances.

Bond type	ZnDTZ	NICS-24	IISERP-MOF36
Zn1−O1	2.0805 (11)	2.13(5)	2.078
Zn1−O2	2.2608 (12)	2.10(5)	2.257
Zn1−N1	2.0591 (12)	2.04(4)	2.049
Zn2−N2	1.9732 (11)	2.02(5)	1.967
Zn2−N3	1.9983 (12)	1.99(4)	1.988
N1-C3	1.354 (2)	1.35 (8)	1.351(6)
O1-C4	1.260 (2)	1.27 (6)	1.263(4)

Table S5 Comparison of selected bond angles.

Atom number			Bond angle		
1	2	3	ZnDTZ	NICS-24	IISERP-MOF36
O1	Zn1	N1	91.79	90.03	91.50
O1	Zn1	O2	76.13	77.82	76.50
O1	Zn1	N1	104.80	104.00	105.10
N2	Zn2	N3	107.04	105.50	107.50
N2	Zn2	N3	108.98	107.50	109.00
N3	Zn2	N3	110.01	110.85	109.60

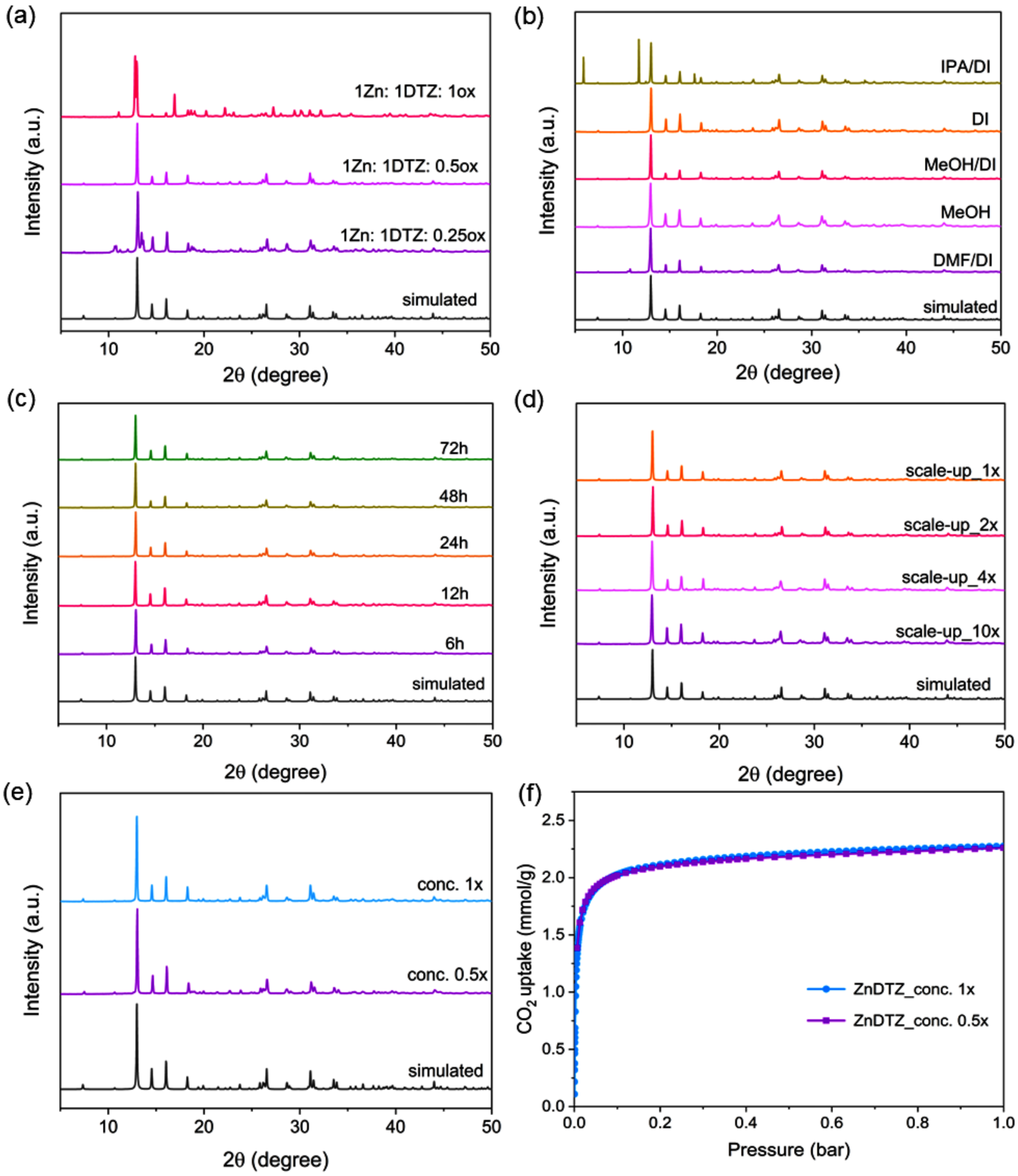

Figure S1. Optimization of reaction conditions for large-scale ZnDTZ MOF synthesis including PXRD patterns obtained under various synthesis conditions: (a) different molar ratio of reactants in MeOH/DI at 423 K for 48 h, (b) different solvents at 423 K for 48 h, (c) varying reaction times in DI water at 363 K, (d) scaling up the reaction in DI water at 363 K for 6 h, and (e) different reaction concentration in DI water at 363 K for 6 h and (f) CO_2 adsorption isotherm at 298 K of samples prepared with different reaction concentrations.

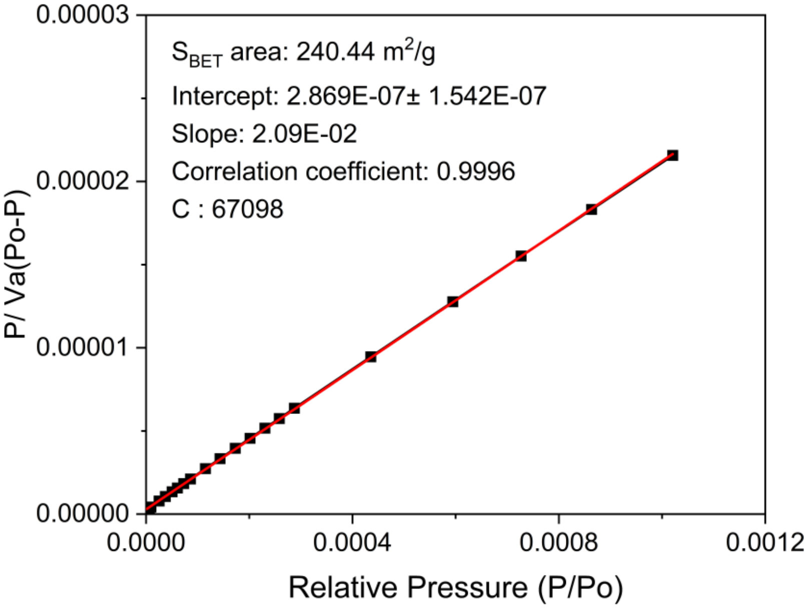

Figure S2. BET fit calculated using the 273 K CO_2 adsorption isotherm.

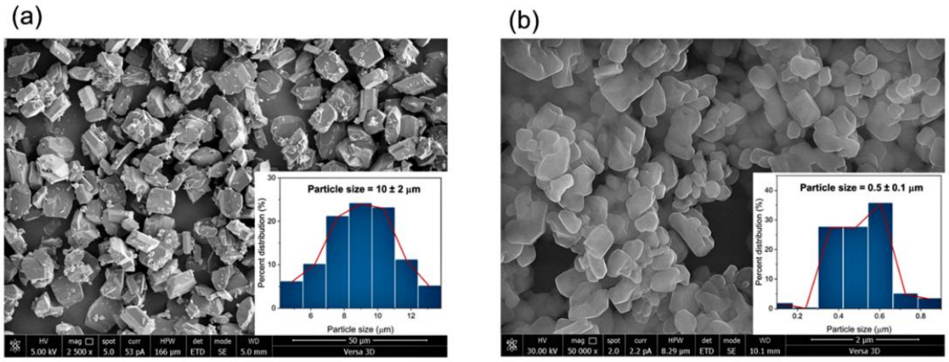

Figure S3. SEM images of (a) ZnDTZ powder and (b) CALF-20.

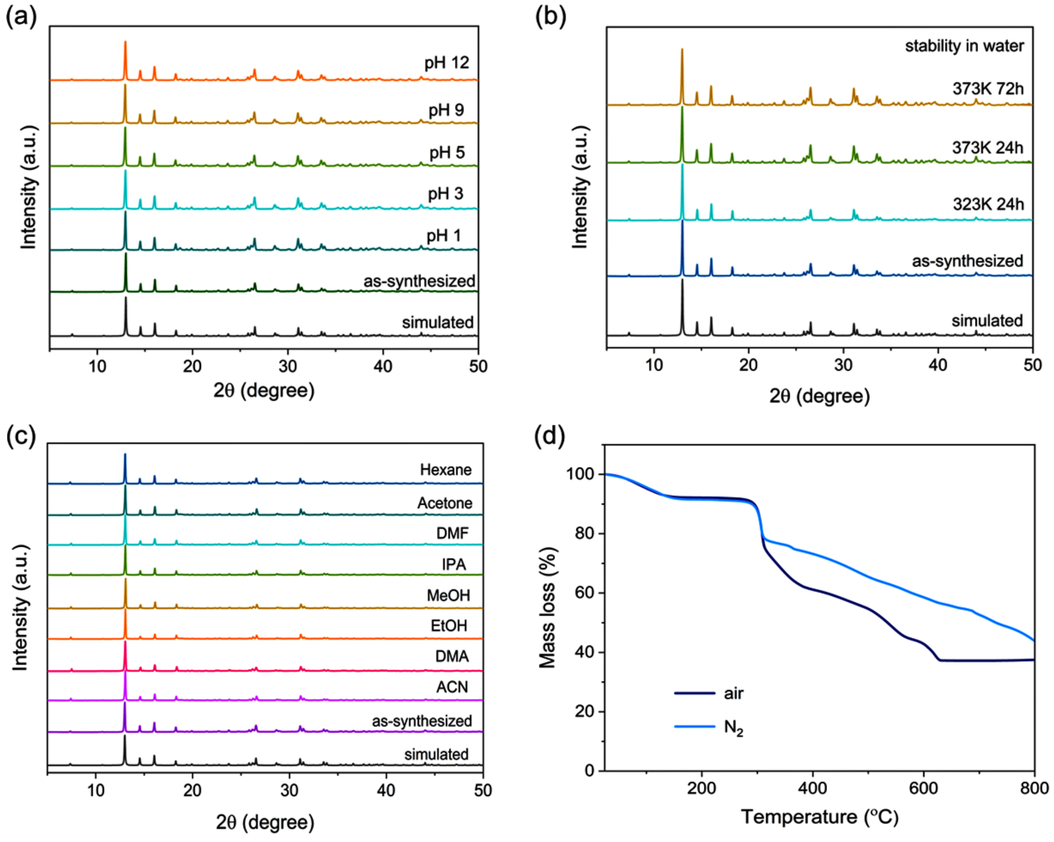

Figure S4. Stability test of as-synthesized ZnDTZ under various conditions. PXRD patterns of (a) ZnDTZ immersed in water for 24 h at room temperature and pH ranging from 1–12, (b) ZnDTZ after heating hot water at different temperatures and durations, (c) ZnDTZ immersed in different organic solvents for 24 h at room temperature, and (d) TGA profiles of ZnDTZ under flow of nitrogen (light blue line) and air (dark blue line) with a ramp rate of 5 K/min.

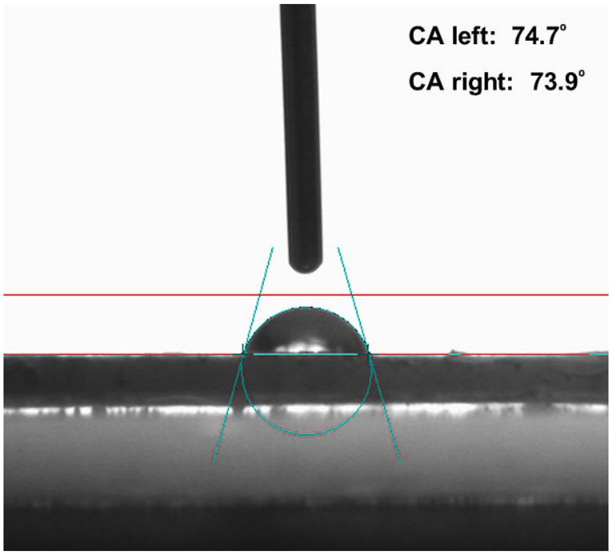

Figure S5. Contact angle measurement for DI water on ZnDTZ.

Section S8. CO_2/N_2 selectivity calculation parameter and results

Parameters

Temp (K)	CO_2			N_2		
	q_{max}^0	k	n	q_{max}^0	k	n
298	2.49334	0.60338	0.74605	2.9582	0.000252	1.01706
303	2.21087	0.53058	0.73136	2.26738	0.000254	1.03873
308	2.08452	0.27048	0.71296	2.47369	0.000192	1.02335
318	1.94833	0.22681	0.7764	3.04769	0.000134	1.01094

1. Adsorption isotherm and ANOVA

1.1. CO_2 adsorption isotherm at 298 K

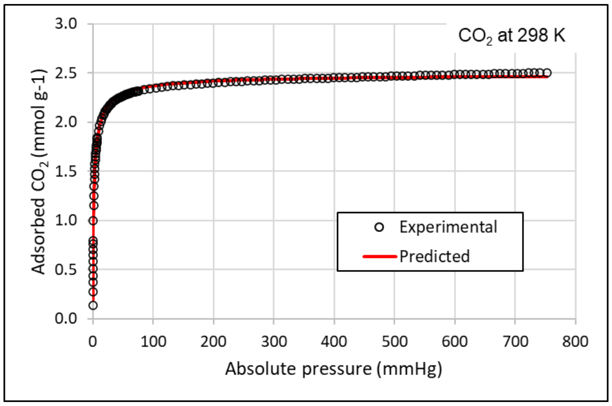

R-Square = 0.9982

	DF	Sum of Squares	Mean Square	F Value	Prob>F
**Regression**	3	648.0625	216.0208	384982.4	<0.0001
**Residual**	132	0.07407	5.61E-04		
**Uncorrected Total**	135	648.1365			
**Corrected Total**	134	40.8838			

1.2. N_2 adsorption isotherm at 298 K

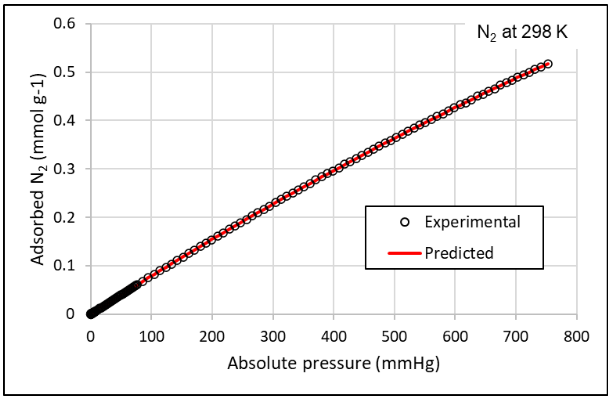

R-Square = 1.00

	DF	Sum of Squares	Mean Square	F Value	Prob>F
**Regression**	3	7.80903	2.60301	26974800	<0.0001
**Residual**	132	1.27E-05	9.65E-08		
**Uncorrected Total**	135	7.80904			
**Corrected Total**	134	3.95996			

1.3. CO_2 adsorption isotherm at 303 K

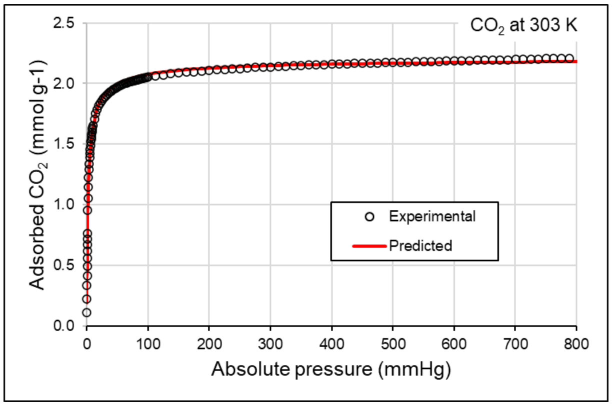

R-Square = 0.9975

	DF	Sum of Squares	Mean Square	F Value	Prob>F
**Regression**	3	648.0625	216.0208	384982.4	<0.0001
**Residual**	132	0.07407	5.61E-04		
**Uncorrected Total**	135	648.1365			
**Corrected Total**	134	40.8838			

1.4. N_2 adsorption isotherm at 303 K

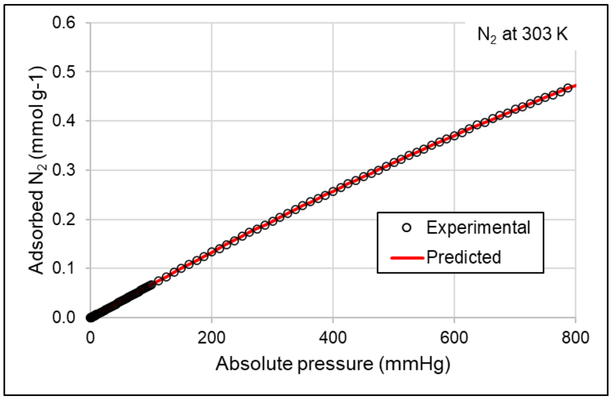

R-Square = 1.00

	DF	Sum of Squares	Mean Square	F Value	Prob>F
**Regression**	3	7.63986	2.54662	20981700	<0.0001
**Residual**	126	1.53E-05	1.21E-07		
**Uncorrected Total**	129	7.63988			
**Corrected Total**	128	3.93277			

1.5. CO_2 adsorption isotherm at 308 K

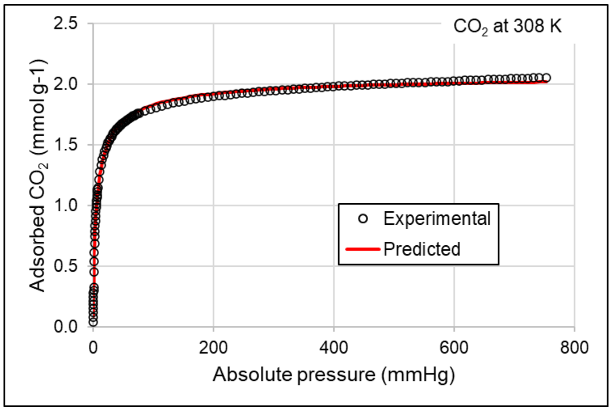

R-Square = 0.9979

	DF	Sum of Squares	Mean Square	F Value	Prob>F
**Regression**	3	383.892	127.964	198986.3	<0.0001
**Residual**	132	0.08489	6.43E-04		
**Uncorrected Total**	135	383.9768			
**Corrected Total**	134	40.48504			

1.6. N_2 adsorption isotherm at 308 K

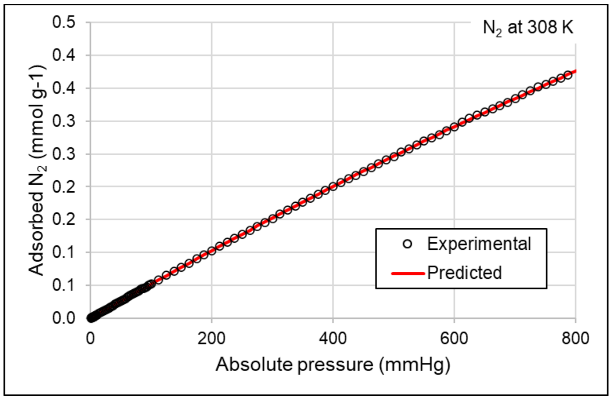

R-Square = 1.00

	DF	Sum of Squares	Mean Square	F Value	Prob>F
**Regression**	3	4.76956	1.58985	4223411	<0.0001
**Residual**	126	4.74E-05	3.76E-07		
**Uncorrected Total**	129	4.76961			
**Corrected Total**	128	2.47503			

1.7. CO_2 adsorption isotherm at 318 K

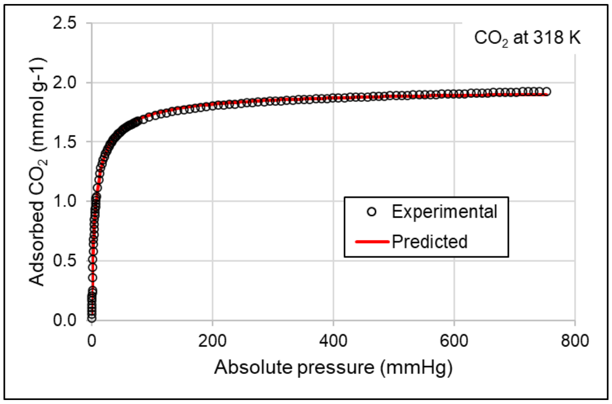

R-Square = 0.9988

	DF	Sum of Squares	Mean Square	F Value	Prob>F
**Regression**	3	340.4928	113.4976	323024	<0.0001
**Residual**	132	0.04638	3.51E-04		
**Uncorrected Total**	135	340.5392			
**Corrected Total**	134	38.39793			

1.8. N_2 adsorption isotherm at 318 K

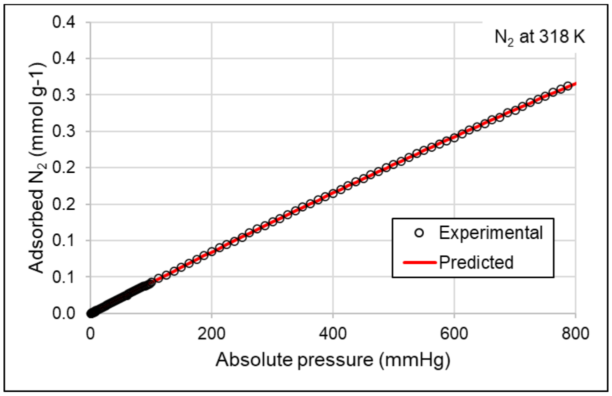

R-Square = 1.00

	DF	Sum of Squares	Mean Square	F Value	Prob>F
**Regression**	3	3.33382	1.11127	4751900	<0.0001
**Residual**	126	2.95E-05	2.34E-07		
**Uncorrected Total**	129	3.33385			
**Corrected Total**	128	1.74133			

2. CO_2/N_2 selectivity results

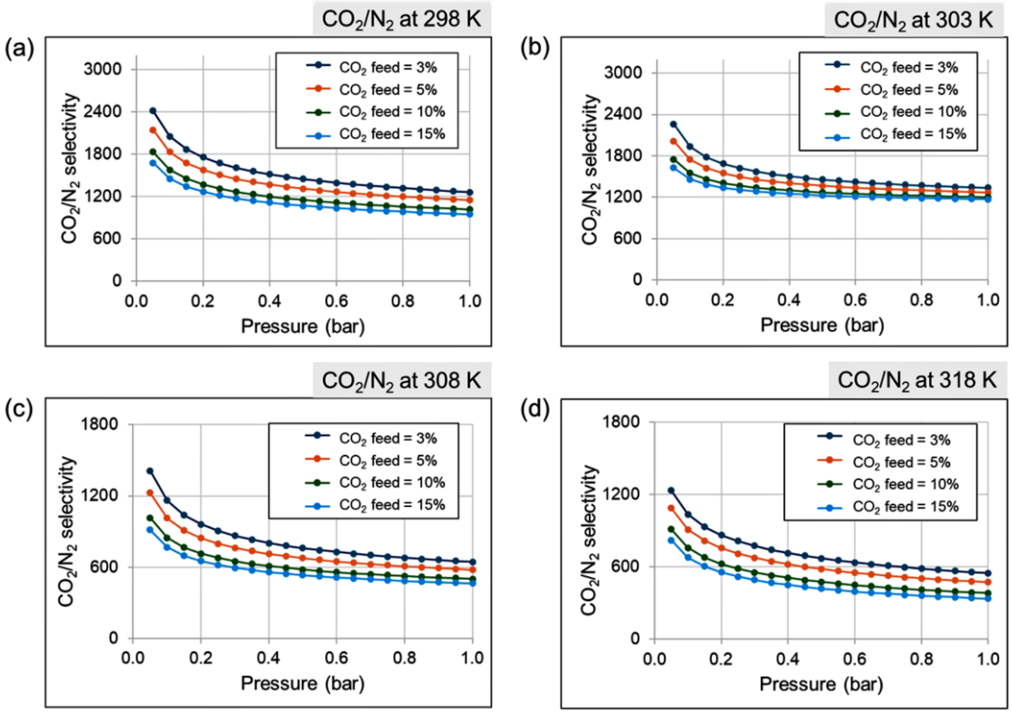

Figure S6. CO_2/N_2 selectivity of ZnDTZ as a function of pressure for four CO_2 molar ratio of inlet mixed gas at (a) 298 K, (b) 303 K, (c) 308 K , and (d) 318 K.

Table S6. Summary of CO_2 adsorption properties and isosteric heat of adsorption (Qst) of various MOFs.

MOFs adsorbent	Temperature (K)	CO_2 uptake (mmol/g)		Qst (kJ/mol)	Main mechanisms	Ref.
		0.05 bar	0.15 bar			
ZnDTZ	298	2.22	2.36	−50.4	physisorption	This work
CALF-20	293	2.6	2.8	−39.0	physisorption	[10]
SIFSIX-3-Ni	298	2.4	2.6	−50.9	kinetic sieving/Lewis interaction	[11]
mmen-Mg_2(dobpdc)	298	2.9	3.1	−71.0	OMSs	[12]
Mg-MOF-74	296	4.5	5.8	−47.0	OMSs	[13]
MIL-120(Al)	298	1.2	2.0	−44.0	pore confinement	[14]
UTSA-16	296	2.0	4.2	−34.6	OMSs	[15]
$Zn_2(Atz)_2$(ox) (CALF-15)	293	1.2	2.1	−40.8	physisorption	[16]
IISERP-MOF28	298	1.3	2.2	−32.0	physisorption	[17]
ZnF(daTZ)	298	0.9	1.8	−33.0	amino functionality	[18]
Cu(adci)-2	298	1.0	2.01	−27.5	OMSs	[19]
MUF-16	293	0.7	1.3	−32.3	physisorption and pore size	[20]
COK-17	298	0.7	1.22	−28.5	van der Waals interactions	[21]
AlF	298	1.4	2.7	−47.9	physisorption	[22]

Section S9. *In situ* DRIFTS of CO_2 adsorption of ZnDTZ and CALF-20 result

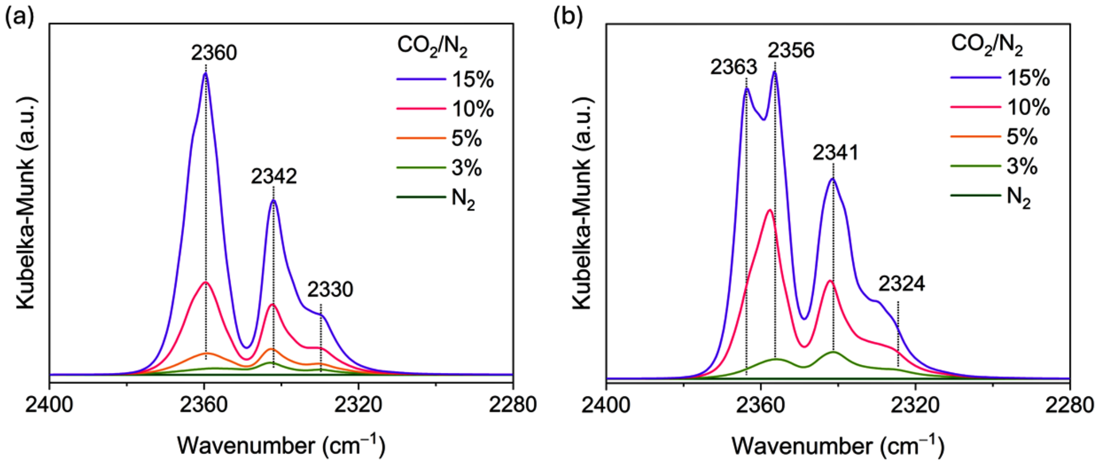

Figure S7. *In situ* DRIFTs spectra at different CO_2 concentrations in the range of 2280 – 2400 cm^{-1} of (a) ZnDTZ and (b) CALF-20.

Section S10. DFT and GCMC calculation results

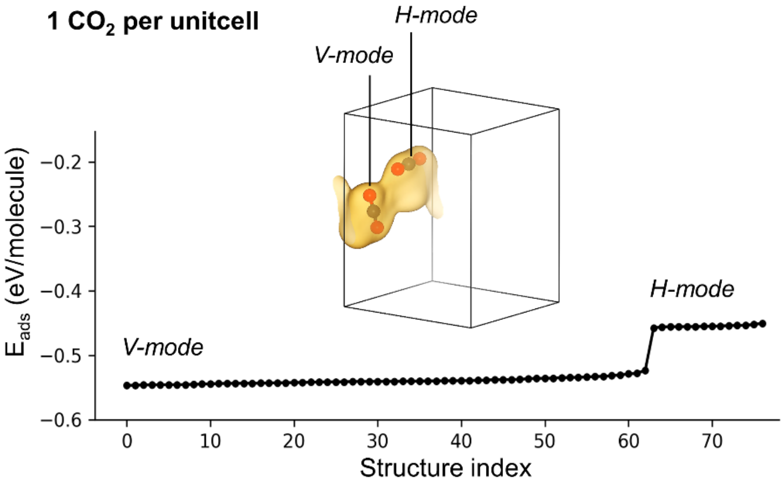

Figure S8. The stable binding configurations in the channel from DFT optimizations using minima hopping method for local minima searching.

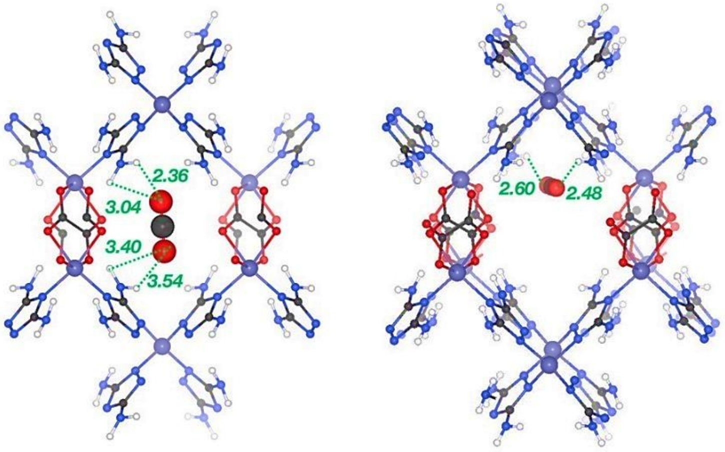

Figure S9. The V–mode (left) and H–mode (right) for the binding configuration of a single CO_2 molecule in the large pore of ZnDTZ. In both binding modes, the primary interaction between the $–NH_2$ hydrogen and the oxalate oxygen is clearly observed.

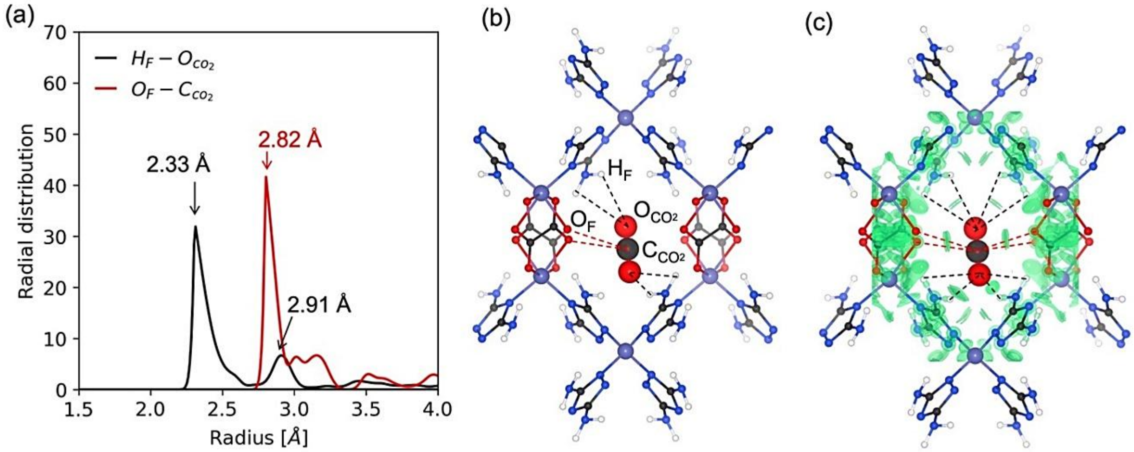

Figure S10. (a) The radial distribution function (RDF) evaluated between the hydrogen atoms of the $–NH_2$ group in ZnDTZ and the oxygen atoms of CO_2 (black line), as well as between the oxygen atoms of oxalate and the carbon atoms of CO_2 (red line). The RDF was calculated based on the average structure obtained from minima hopping at the DFT level. (b) The label of atoms involved in RDF. (c) The non-covalent interaction analysis using the density overlap regions indicator (DORI) method showing the non-covalent bond interaction between $–NH_2$ and CO_2 oxygen, and between oxalate oxygen and CO_2 carbon (isosurface DORI = 0.98) (C: black, O: red, Zn: purple, H: white).

Table S7. The vibrational frequencies for N–H stretches for $–NH_2$ estimated from DFT level of theory. Noted that the absolute values are known for the deviation from the experiment due to level of theory, therefore the response of vibrations in different environments were compared. The V mode for single CO_2 adsorption represents a low-concentration scenario where no neighboring CO_2 molecules are present. In contrast, the V–V mode for two CO_2 adsorptions corresponds to a high-concentration scenario, where adjacent CO_2 molecules occupy neighboring sites.

**vibrational mode**	**$–NH_2$ type**	**ZnDTZ**	**1 CO_2**		**2 CO_2**		
		**clean**	**V mode**	**H mode**	**V–V mode**	**V–H mode**	**H–H mode**
Symmetric N–H stretches	Small pore	**3385.9**	**3389.1**	3376.9	**3395.0**	3381.2	3375.4
		**3390.5**	**3393.4**	3383.5	**3398.7**	3387.0	3379.4
		**3391.3**	**3396.4**	3386.4	**3403.0**	3396.2	3392.9
		**3392.5**	**3399.7**	3394.8	**3408.1**	3399.2	3395.8
	Large pore	**3473.5**	**3472.0**	3475.8	**3471.1**	3474.8	3472.6
		**3475.4**	**3477.5**	3477.6	**3472.8**	3476.6	3474.8
		**3475.8**	**3479.4**	3478.5	**3477.9**	3478.6	3476.3
		**3476.8**	**3480.8**	3483.6	**3479.4**	3480.8	3477.3
Asymmetric N–H stretches	Small pore	**3524.1**	**3526.0**	3523.3	**3527.6**	3521.0	3524.5
		**3526.0**	**3530.1**	3525.2	**3529.8**	3528.5	3527.2
		**3526.1**	**3532.0**	3527.9	**3530.5**	3530.9	3529.9
		**3527.6**	**3534.9**	3531.9	**3532.6**	3532.1	3530.5
	Large pore	**3584.0**	**3571.6**	3577.5	**3570.1**	3574.3	3574.4
		**3584.0**	**3576.9**	3578.8	**3571.9**	3578.0	3576.1
		**3584.0**	**3583.6**	3587.8	**3578.8**	3581.0	3577.2
		**3586.0**	**3587.0**	3594.7	**3580.7**	3584.5	3579.1

Table S8. The vibrational frequencies for C–O stretches for CO_2 estimated from DFT level of theory. Noted that the absolute values are known for the deviation from the experiment due to level of theory, therefore the response in different environments were compared. The splitting at V–V mode with 10 cm^{-1} interval is in well agreement with the experiment, confirming that the binding mode of CO_2 in the cavity is dominated by V–V mode configuration.

vibrational mode	CO_2 site	ZnDTZ	1 CO_2		2 CO_2		
		clean	V mode	H mode	V–V mode	V–H mode	H–H mode
C–O stretches	large pore		2355.5	2353.0	**2347.9**	2356.0	2343.6
					**2358.1**	2357.1	2357.6

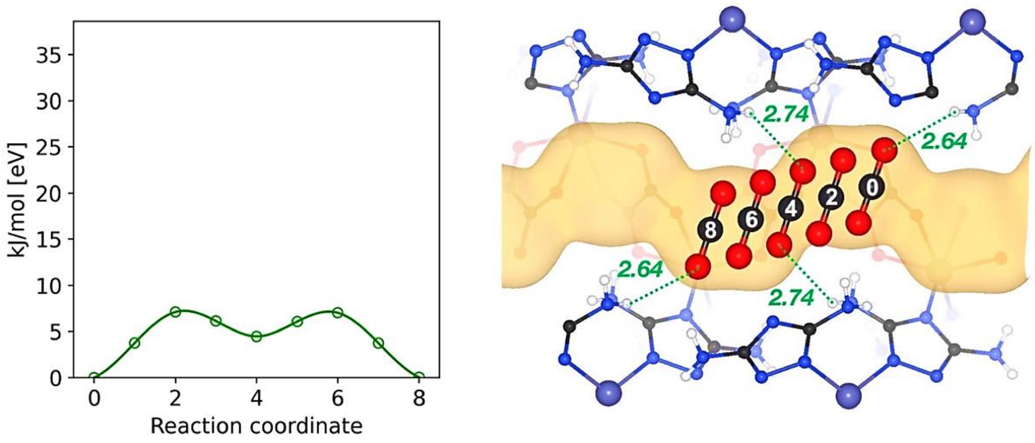

Figure S11. The nudged elastic band (NEB) analysis within the DFT framework for CO_2 hopping between neighboring local minima in the one-dimensional channel of ZnDTZ confirming an almost barrierless migration, with a barrier of only 7.10 kJ/mol (0.0727 eV).

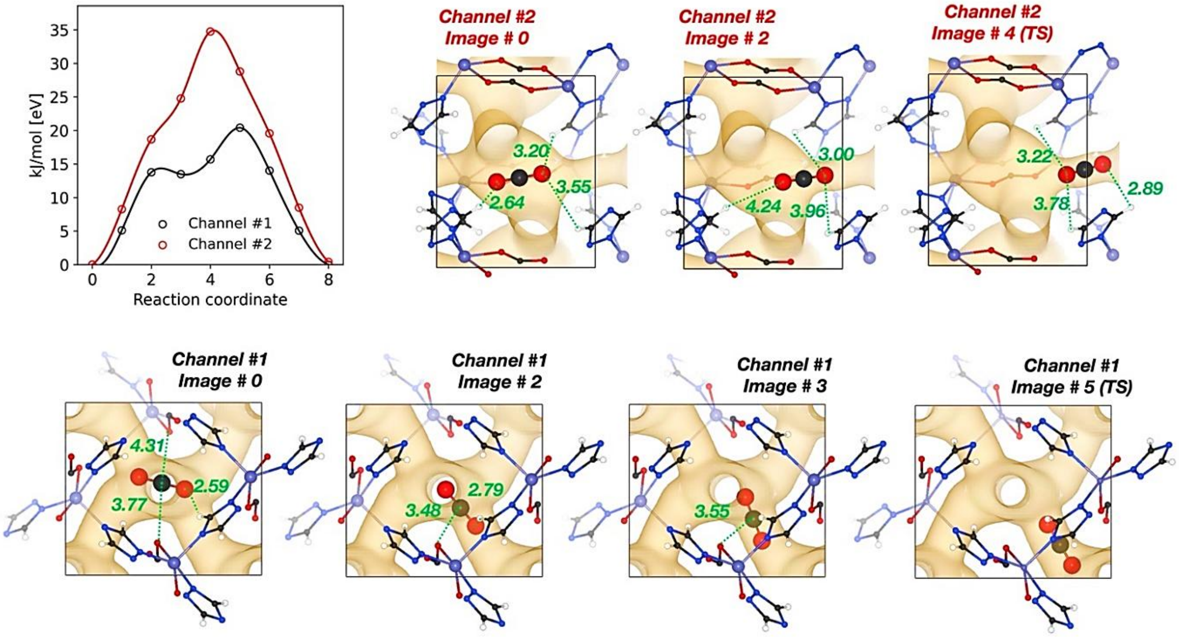

Figure S12. The nudge elastic band (NEB) analysis within DFT framework for CO_2 hopping between local minima in neighboring pores in CALF-20 showing that the migration barriers of 20.06 and 34.74 kJ/mol for channel#1 and channel#2, respectively.

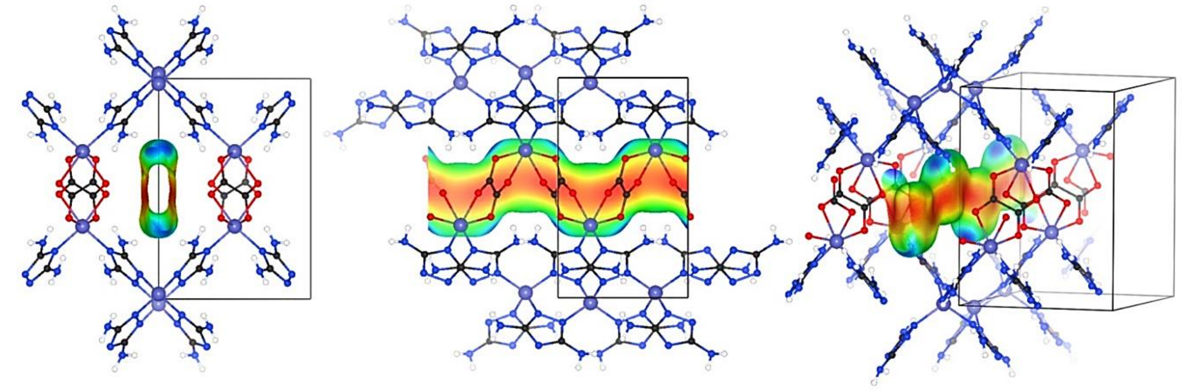

Figure S13. The electrostatic potential is projected onto the one-dimensional wall of ZnDTZ, with red and blue indicating negative and positive electrostatic potentials, respectively. This unique feature, where the field is characterized along a single axis, results in an almost barrierless translation of CO_2.

Section S11. Gravimetric CO_2 adsorption results

Table S9. CO_2 uptake of ZnDTZ and CALF-20 at different temperatures and CO_2 concentrations by volumetric method and TGA/DSC.

Sample name	Volumetric method				Gravimetric method (TGA/DSC)					
	Pure CO_2 uptake (mmol/g)				3% CO_2/N_2, 150 sccm		5% CO_2/N_2, 150 sccm		15% CO_2/N_2, 150 sccm	
	0.03 bar	0.05 bar	0.15 bar	1 bar	CO_2 uptake (mmol/g)	T_{regen} (K)	CO_2 uptake (mmol/g)	T_{regen} (K)	CO_2 uptake (mmol/g)	T_{regen} (K)
ZnDTZ_298 K	2.13	2.22	2.36	2.52	N/A	N/A	N/A	N/A	N/A	N/A
ZnDTZ_303 K	1.89	1.97	2.09	2.21	1.61	376.3	1.73	372.7	1.97	369.3
ZnDTZ_308 K	1.49	1.62	1.82	2.06	1.55	374.6	1.69	374.7	1.93	373.8
ZnDTZ_318 K	1.40	1.53	1.73	1.93	1.40	377.8	1.56	375.3	1.88	375.1
CALF-20_298 K	1.72	2.09	2.71	3.88	1.26	326.2	1.63	329.5	2.39	331.2
CALF-20_303 K	1.13	1.54	2.18	3.66	1.06	313.4	1.43	329.1	2.24	339.1
CALF-20_308 K	N/A	N/A	N/A	N/A	0.87	315.2	1.23	333.0	2.06	339.5
CALF-20_318 K	N/A	N/A	N/A	N/A	0.58	335.4	0.86	337.8	1.73	340.3

T_{regen} = regeneration temperature

Table S10. Comparison of adsorption time at 90% of equilibrium capacity with different CO_2 concentration of 3%, 5%, and 15% CO_2/N_2 of ZnDTZ and CALF-20.

Sample name	Adsorption time (min) at 90% of equilibrium capacity		
	3% CO_2/N_2	5% CO_2/N_2	15% CO_2/N_2
ZnDTZ at 303 K	4.05	3.18	1.47
ZnDTZ at 308 K	3.80	3.08	1.38
ZnDTZ at 318 K	3.70	2.85	1.37
CALF-20 at 303 K	6.47	5.92	3.43
CALF-20 at 308 K	5.67	5.85	2.93
CALF-20 at 318 K	4.53	4.37	2.90

Section S12. Fitting curve of pseudo first order (PFO) and pseudo second order (PSO) kinetic models

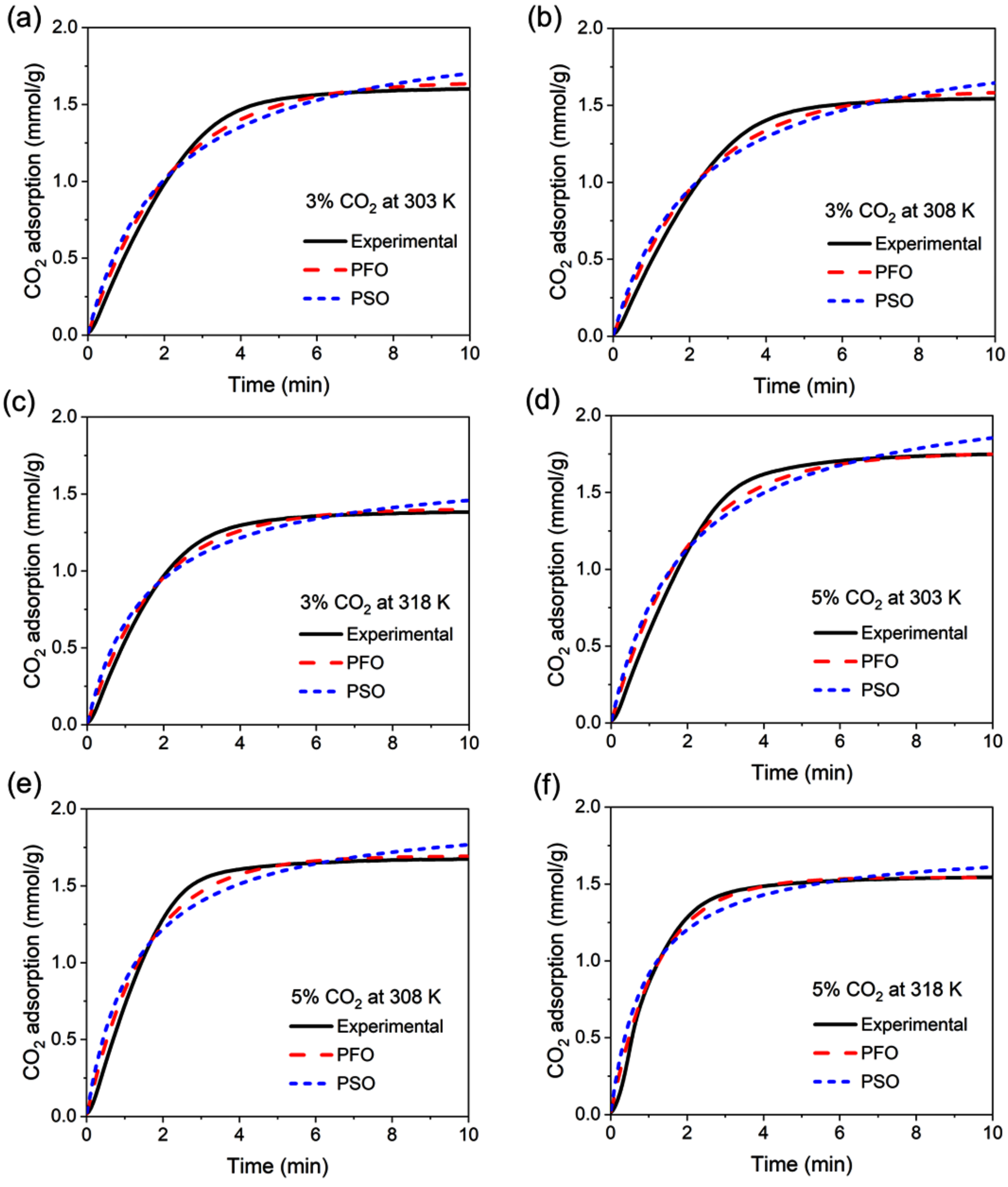

Figure S14. Fittings for pseudo first order (PFO) and pseudo second order (PSO) kinetic models for ZnDTZ at the conditions of 3% CO_2 at (a) 303 K (b) 308 K and (c) 318 K, for 5% CO_2 at (d) 303 K (e) 308 K and (f) 318 K and for 15% CO_2 at (g) 303 K (h) 308 K and (i) 318 K.

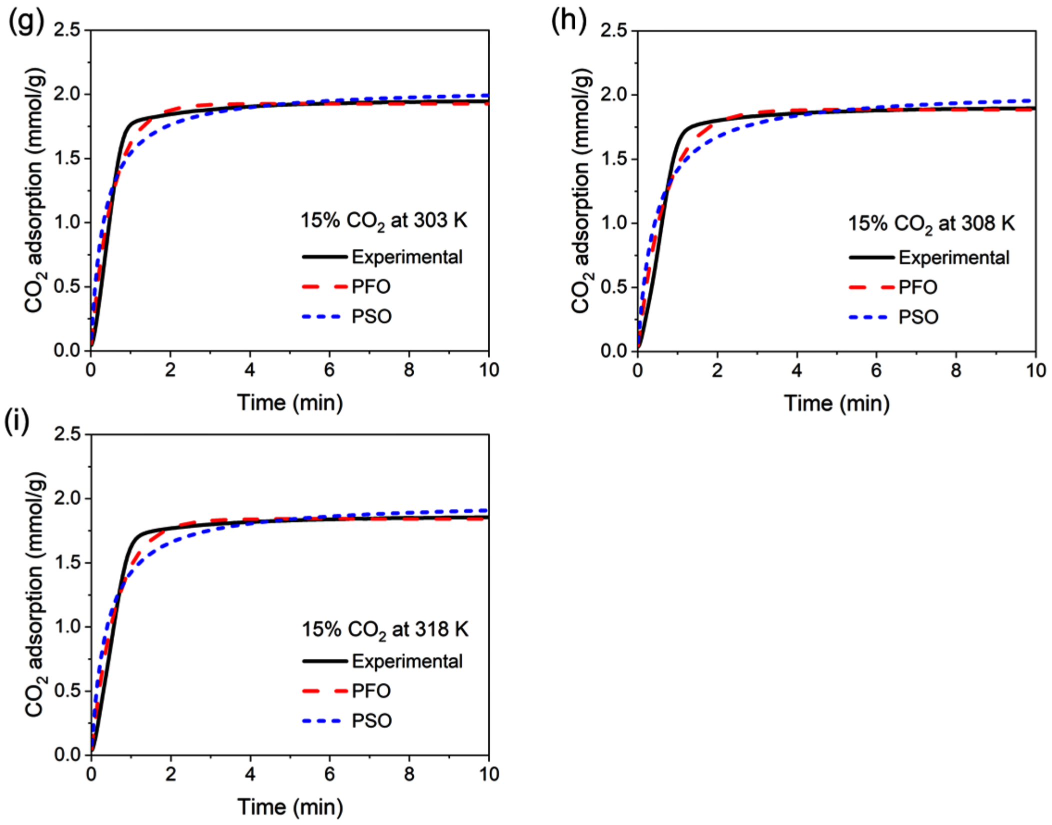

Figure S14 (cont'd). Fittings for pseudo first order (PFO) and pseudo second order (PSO) kinetic models for ZnDTZ at the conditions of 3% CO_2 at (a) 303 K (b) 308 K and (c) 318 K, for 5% CO_2 at (d) 303 K (e) 308 K and (f) 318 K, and for 15% CO_2 at (g) 303 K (h) 308 K and (i) 318 K.

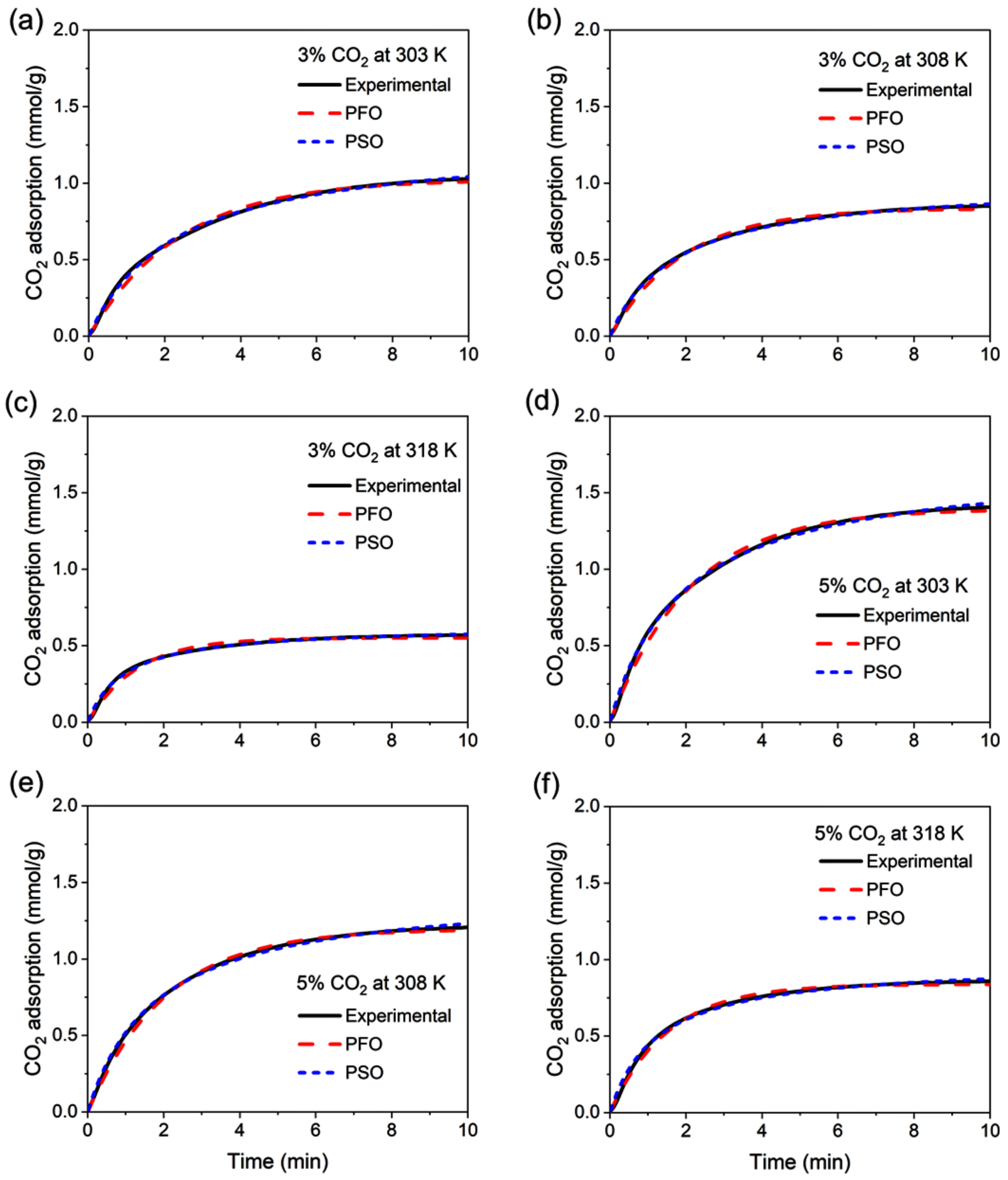

Figure S15. Fittings for pseudo first order (PFO) and pseudo second order (PSO) kinetic models for CALF-20 at the conditions of 3% CO_2 at (a) 303 K (b) 308 K and (c) 318 K, for 5% CO_2 at (d) 303 K (e) 308 K and (f) 318 K, and for 15% CO_2 at (g) 303 K (h) 308 K and (i) 318 K.

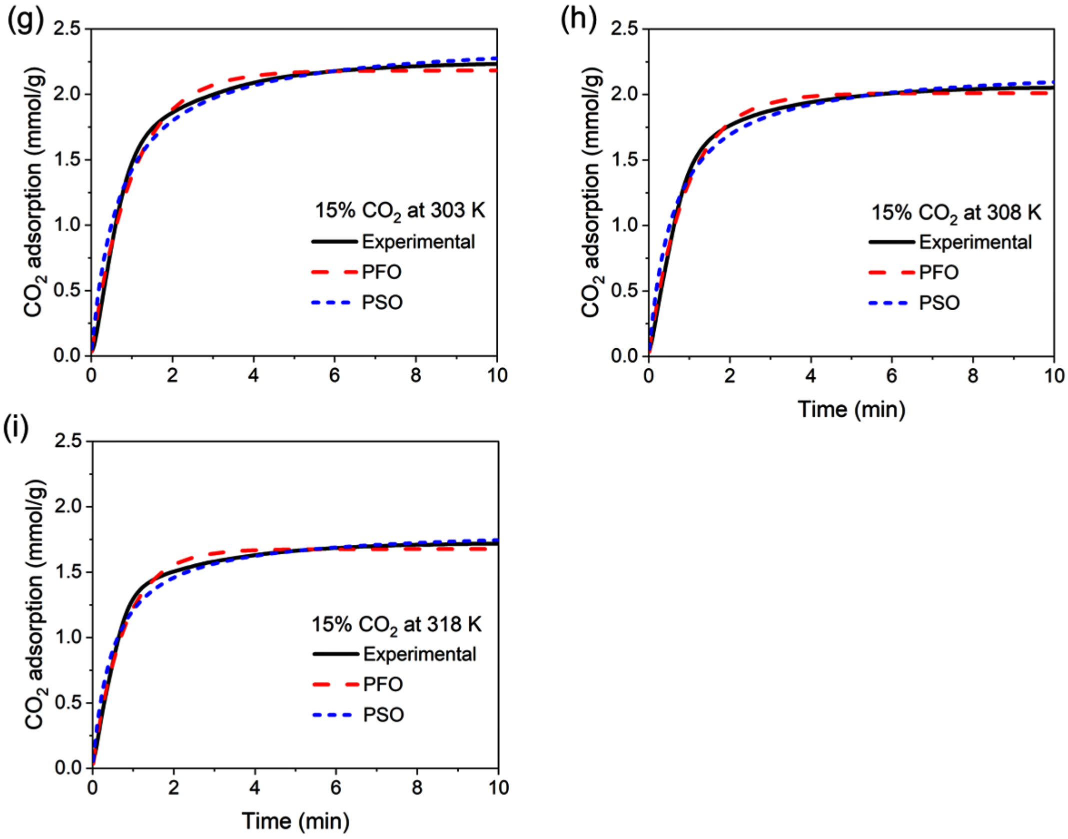

Figure S15 (cont'd). Fittings for pseudo first order (PFO) and pseudo second order (PSO) kinetic models for CALF-20 at the conditions of 3% CO_2 at (a) 303 K (b) 308 K and (c) 318 K, for 5% CO_2 at (d) 303 K (e) 308 K and (f) 318 K, and for 15% CO_2 at (g) 303 K (h) 308 K and (i) 318 K.

The diffusion coefficient could be expressed by the following equation describing the fractional CO_2 uptake and the crystal radius and time of adsorption.[23, 24]

$$\frac{q_t}{q_e} = \frac{6}{r_c}\sqrt{\frac{D_M t}{\pi}} \qquad \text{Equation S13}$$

The diffusion coefficient was extracted from the gravimetric adsorption data by plotting the fractional CO_2 uptake (q_t/q_e) against the square root of the adsorption time. The slope of the plot could be derived as the diffusion time constant ($D_M/r\mathrm{c}^2$) could be derived to calculate the intracrystalline diffusion coefficient (D_M). The crystal radius (r_c^2) was obtained from the particle size distribution from the SEM images (Figure S2).

Table S11 Micropore diffusion fitting parameter for ZnDTZ and CALF-20 at different CO_2 adsorption conditions. It is important to note that the intracrystalline diffusion coefficient is a function of particle size which can significantly influence the estimated absolute values.

CO_2 concentration (%)	Temperature (K)	Intracrystalline Diffusion Coefficient, D_M (10^{-9} cm^2 s^{-1})	
		ZnDTZ	CALF-20
3	303	18.825	0.037
	308	15.550	0.041
	318	18.825	0.048
5	303	19.925	0.040
	308	20.600	0.041
	318	23.525	0.052
15	303	34.400	0.047
	308	33.825	0.048
	318	33.855	0.045

Table S12. Comparison of CO_2 diffusion coefficients for ZnDTZ and other materials. It should be noted that these values are presented to provide a landscape of the diffusivity values reported in the literature. However, they can vary significantly, as they are derived from samples from samples with differing particle sizes and obtained using various measurement methods and experimental conditions. These discrepancies can substantially affect absolute values.

Adsorbent	Measurement method, CO_2 concentration	Temperature (K)	D_M (10^{-9} cm^2 s^{-1})	Ref.
ZnDTZ	gravimetric, 5%CO_2	303	19.925	This work
CALF-20	gravimetric, 5%CO_2	303	0.040	This work
MOF-5	gravimetric, 50%CO_2	296	7.9	[23]
Zeolite 13X	manometry, 100%CO_2	303	0.0649	[25]
Granular Activated carbon	volumetric, 100%CO_2	298	0.578	[24]
CID-Me	NMR	298	0.01–1	[26]
Zeolite 4A pellet	gravimetric	303	0.001	[27]

Table S13. Pseudo first and second order kinetics model parameters of CO_2 adsorption at different adsorption conditions for ZnDTZ and CALF-20.

**Model**	**Kinetics**		**ZnDTZ**			**CALF-20**		
	CO_2	Parameter	303 K	308 K	318 K	303 K	308 K	318 K
Pseudo 1st order (PFO)	3 %	k_1 (1/min)	0.47	0.45	0.58	0.42	0.53	0.79
		q_e (exp)	1.61	1.60	1.40	1.06	0.87	0.58
		q_e (calc)	1.65	1.55	1.40	1.02	0.84	0.55
		SSE (%)	4.61	4.68	3.24	2.09	1.72	1.66
	5 %	k_1 (1/min)	0.50	0.66	0.83	0.48	0.49	0.66
		q_e (exp)	1.76	1.69	1.56	1.43	1.23	0.86
		q_e (calc)	1.79	1.69	1.54	1.39	1.20	0.84
		SSE (%)	5.35	5.00	3.01	2.43	1.43	1.58
	15 %	k_1 (1/min)	1.83	1.47	1.61	1.00	1.09	1.32
		q_e (exp)	1.97	1.93	1.88	2.24	2.06	1.73
		q_e (calc)	1.93	1.89	1.84	2.18	2.01	1.68
		SSE (%)	5.54	6.37	5.44	4.74	3.80	3.83
Pseudo 2nd order (PSO)	3 %	k_2 (mmol/g min)	0.24	0.22	0.38	0.34	0.58	1.68
		q_e (exp)	1.61	1.55	1.40	1.06	0.87	0.58
		q_e (calc)	2.05	2.01	1.68	1.28	1.01	0.63
		SSE (%)	8.01	7.83	6.61	0.86	0.65	0.60
		E_a (kJ/mol)		49.85			40.82	
		R^2 (E_a)		0.8905			0.9988	
	5 %	k_2 (mmol/g min)	0.24	0.40	0.62	0.31	0.38	0.84
		q_e (exp)	1.76	1.69	1.56	1.43	1.23	0.86
		q_e (calc)	2.21	1.99	1.76	1.71	1.45	0.98
		SSE (%)	9.16	9.35	7.09	1.35	1.17	1.23
		E_a (kJ/mol)		40.98			26.60	
		R^2 (E_a)		0.9999			0.9841	
	15 %	k_2 (mmol/g min)	1.45	1.11	1.30	0.58	0.71	1.06
		q_e (exp)	1.97	1.93	1.88	2.24	2.06	1.73
		q_e (calc)	2.06	2.04	1.98	2.44	2.23	1.83
		SSE (%)	9.69	11.02	9.99	5.87	5.66	4.54
		E_a (kJ/mol)		30.95			15.55	
		R^2 (E_a)		0.9977			1.0000	

Section S13. Recyclability results

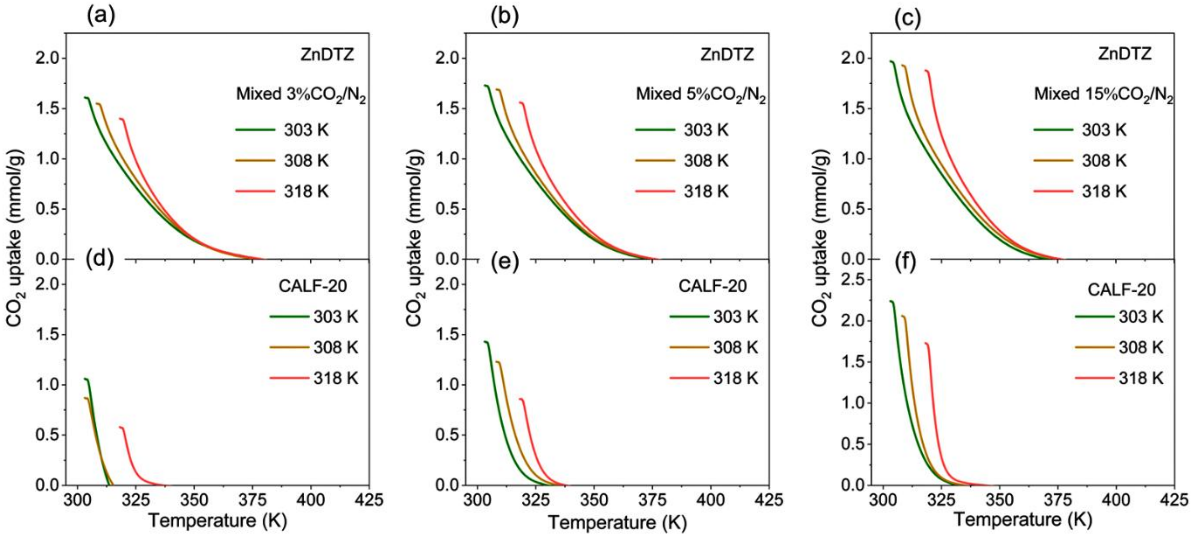

Figure S16. CO_2 Desorption profiles of (a, b, c) ZnDTZ and (d, e, f) of CALF-20 for dosing of mixed 3% CO_2/N_2, 5% CO_2/N_2, and 15% CO_2/N_2 at 303–318 K, respectively. Condition: ramp rate of 10 K/min from 303–423 K under N_2 without holding time.

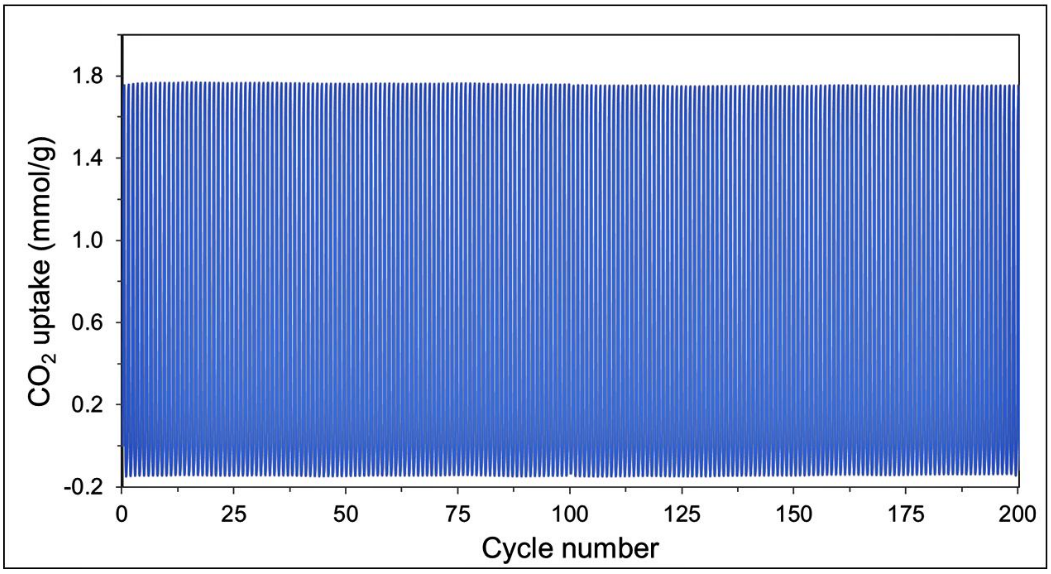

Figure S17. Recyclability test of ZnDTZ under temperature swing conditions between CO_2 adsorption at 303 K, 5% CO_2/N_2, 10 min and desorption at 423 K under N_2 at ramp rate of 10 K/min.

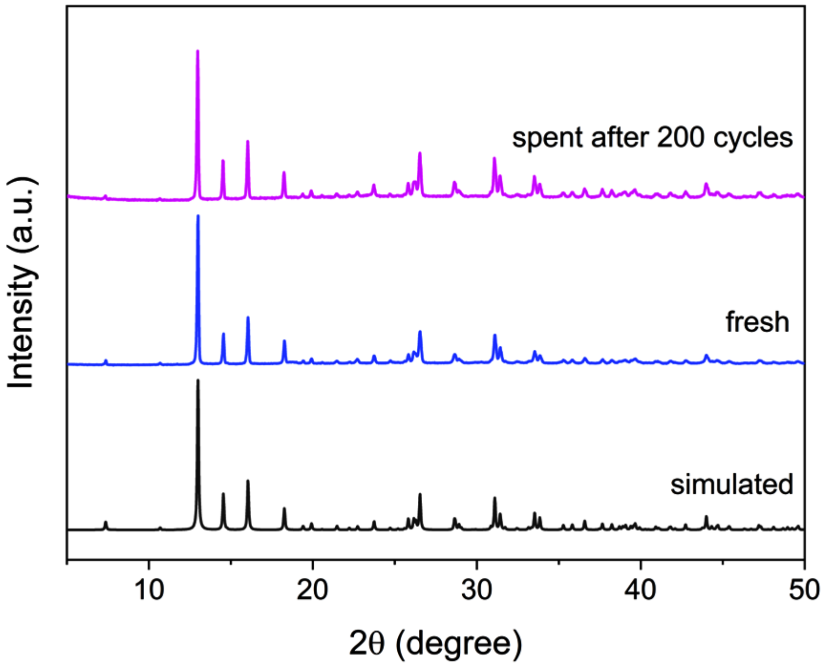

Figure S18. PXRD pattern of fresh and spent ZnDTZ after 200 cycles of temperature-swing of CO_2 adsorption-desorption cycles.

Section S14. Dynamic breakthrough results

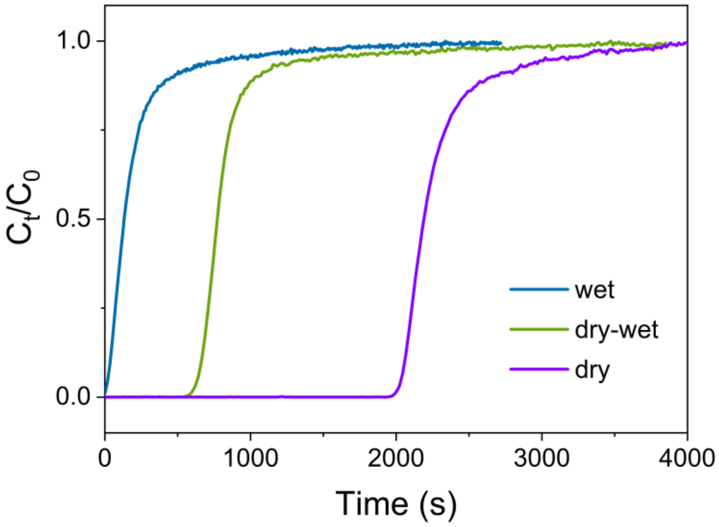

Figure S19. Competitive CO_2 and H_2O adsorption dynamic breakthrough curves of ZnDTZ at 303 K under three conditions: dry (5% CO_2/N_2, purple line), dry-wet (5% $CO_2/N_2/H_2O$, 40% RH, green line), and wet (40% RH, H_2O/N_2 and then 5% $CO_2/N_2/H_2O$, 40% RH, blue line).

REFERENCES

(1) APEX5, SADABS and SAINT. Bruker AXS Inc.: Madison, Wisconsin, USA, 2023.

(2) Sheldrick, G., SHELXT - Integrated space-group and crystal-structure determination. *Acta Crystallogr. A: Found. Adv.* **2015,** *71* (1), 3-8.

(3) Sheldrick, G., Crystal structure refinement with SHELXL. *Acta Cryst. C* **2015,** *71* (1), 3-8.

(4) Krishna, R.; van Baten, J. M., How Reliable Is the Ideal Adsorbed Solution Theory for the Estimation of Mixture Separation Selectivities in Microporous Crystalline Adsorbents? *ACS Omega* **2021,** *6* (23), 15499-15513.

(5) Myers, A. L.; Prausnitz, J. M., Thermodynamics of mixed-gas adsorption. *AIChE Journal* **1965,** *11* (1), 121-127.

(6) Cessford, N. F.; Seaton, N. A.; Düren, T., Evaluation of Ideal Adsorbed Solution Theory as a Tool for the Design of Metal–Organic Framework Materials. *Ind. Eng. Chem. Res.* **2012,** *51* (13), 4911-4921.

(7) Tkachenko, O.; Diment, D.; Rigo, D.; Strømme, M.; Budnyak, T. M., Unveiling the Nature of lignin's Interaction with Molecules: A Mechanistic Understanding of Adsorption of Methylene Blue Dye. *Biomacromolecules* **2024,** *25* (7), 4292-4304.

(8) Querejeta, N.; Rubiera, F.; Pevida, C., Experimental Study on the Kinetics of CO_2 and H_2O Adsorption on Honeycomb Carbon Monoliths under Cement Flue Gas Conditions. *ACS Sustain. Chem. Eng.* **2022,** *10* (6), 2107-2124.

(9) Singh, V. K.; Kumar, E. A., Comparative Studies on CO_2 Adsorption Kinetics by Solid Adsorbents. *Energy Procedia* **2016,** *90*, 316-325.

(10) Lin, J.-B.; Nguyen, T. T. T.; Vaidhyanathan, R.; Burner, J.; Taylor, J. M.; Durekova, H.; Akhtar, F.; Mah, R. K.; Ghaffari-Nik, O.; Marx, S.; Fylstra, N.; Iremonger, S. S.; Dawson, K. W.; Sarkar, P.; Hovington, P.; Rajendran, A.; Woo, T. K.; Shimizu, G. K. H., A scalable metal-organic framework as a durable physisorbent for carbon dioxide capture. *Science* **2021,** *374* (6574), 1464-1469.

(11) Chen, K.-J.; Scott, Hayley S.; Madden, David G.; Pham, T.; Kumar, A.; Bajpai, A.; Lusi, M.; Forrest, Katherine A.; Space, B.; Perry, John J.; Zaworotko, Michael J., Benchmark C_2H_2/CO_2 and CO_2/C_2H_2 Separation by Two Closely Related Hybrid Ultramicroporous Materials. *Chem* **2016,** *1* (5), 753-765.

(12) McDonald, T. M.; Lee, W. R.; Mason, J. A.; Wiers, B. M.; Hong, C. S.; Long, J. R., Capture of Carbon Dioxide from Air and Flue Gas in the Alkylamine-Appended Metal–Organic Framework mmen-Mg_2(dobpdc). *J. Am. Chem. Soc.* **2012,** *134* (16), 7056-7065.

(13) Caskey, S. R.; Wong-Foy, A. G.; Matzger, A. J., Dramatic Tuning of Carbon Dioxide Uptake via Metal Substitution in a Coordination Polymer with Cylindrical Pores. *J. Am. Chem. Soc.* **2008,** *130* (33), 10870-10871.

(14) Loughran, R. P.; Hurley, T.; Gładysiak, A.; Chidambaram, A.; Khivantsev, K.; Walter, E. D.; Graham, T. R.; Reardon, P.; Szanyi, J.; Fast, D. B.; Miller, Q. R. S.; Park, A.-H. A.; Stylianou, K. C., CO_2 capture from wet flue gas using a water-stable and cost-effective metal-organic framework. *Cell Rep.* **2023,** *4* (7).

(15) Xiang, S.; He, Y.; Zhang, Z.; Wu, H.; Zhou, W.; Krishna, R.; Chen, B., Microporous metal-organic framework with potential for carbon dioxide capture at ambient conditions. *Nat. Commun.* **2012,** *3* (1), 954.

(16) Vaidhyanathan, R.; Iremonger, S. S.; Dawson, K. W.; Shimizu, G. K. H., An amine-functionalized metal organic framework for preferential CO_2 adsorption at low pressures. *Chem. Commun.* **2009,** (35), 5230-5232.

(17) Singh, P.; Singh, H. D.; Menon, A. H.; Vaidhyanathan, R., Preferential CO_2 adsorption by an ultra-microporous zinc-aminotriazolato-acetate MOF. *Chem. Commun.* **2023,** *59* (37), 5559-5562.

(18) Shi, Z.; Tao, Y.; Wu, J.; Zhang, C.; He, H.; Long, L.; Lee, Y.; Li, T.; Zhang, Y.-B., Robust Metal–Triazolate Frameworks for CO_2 Capture from Flue Gas. *J. Am. Chem. Soc.* **2020,** *142* (6), 2750-2754.

(19) Jo, D.; Lee, S.-K.; Cho, K. H.; Yoon, J. W.; Lee, U. H., An Amine-Functionalized Ultramicroporous Metal–Organic Framework for Postcombustion CO_2 Capture. *ACS Appl. Mater. Interfaces.* **2022,** *14* (51), 56707-56714.

(20) Qazvini, O. T.; Telfer, S. G., MUF-16: A Robust Metal–Organic Framework for Pre- and Post-Combustion Carbon Dioxide Capture. *ACS Appl. Mater. Interfaces.* **2021,** *13* (10), 12141-12148.

(21) Wee, L. H.; Vandenbrande, S.; Rogge, S. M. J.; Wieme, J.; Asselman, K.; Jardim, E. O.; Silvestre-Albero, J.; Navarro, J. A. R.; Van Speybroeck, V.; Martens, J. A.; Kirschhock, C. E. A., Chlorination of a Zeolitic-Imidazolate Framework Tunes Packing and van der Waals Interaction of Carbon Dioxide for Optimized Adsorptive Separation. *J. Am. Chem. Soc.* **2021,** *143* (13), 4962-4968.

(22) Evans, H. A.; Mullangi, D.; Deng, Z.; Wang, Y.; Peh, S. B.; Wei, F.; Wang, J.; Brown, C. M.; Zhao, D.; Canepa, P.; Cheetham, A. K., Aluminum formate, $Al(HCOO)_3$: An earth-abundant, scalable, and highly selective material for CO_2 capture. *Sci. Adv.* **2022,** *8* (44), eade1473.

(23) Zhao, Z.; Li, Z.; Lin, Y. S., Adsorption and Diffusion of Carbon Dioxide on Metal−Organic Framework (MOF-5). *Ind. Eng. Chem. Res.* **2009,** *48* (22), 10015-10020.

(24) Zhang, B.; Liu, P.; Huang, Z.; Liu, J., Adsorption Equilibrium and Diffusion of CH_4, CO_2, and N_2 in Coal-Based Activated Carbon. *ACS Omega* **2023,** *8* (11), 10303-10313.

(25) Kamiuto, K.; Asami, G.; and, E., Diffusion Coefficients of carbon dioxide within type 13X zeolite particles. *Chem. Eng. Commun.* **2006,** *193* (5), 628-638.

(26) Kurihara, T.; Inukai, M.; Mizuno, M., Slow CO_2 Diffusion Governed by Steric Hindrance of Rotatory Ligands in Small Pores of a Metal–Organic Framework. *Phys. Chem. Lett.* **2022,** *13* (30), 7023-7028.

(27) Ahn, H.; Moon, J.-H.; Hyun, S.-H.; Lee, C.-H., Diffusion Mechanism of Carbon Dioxide in Zeolite 4A and CaX Pellets. *Adsorption* **2004,** *10* (2), 111-128.
